# Structural analysis of P. falciparum KAHRP and PfEMP1 complexes with host erythrocyte spectrin suggests a model for cytoadherent knob protrusions

**DOI:** 10.1371/journal.ppat.1006552

**Published:** 2017-08-14

**Authors:** Erin E. Cutts, Niklas Laasch, Dirk M. Reiter, Raphael Trenker, Leanne M. Slater, Phillip J. Stansfeld, Ioannis Vakonakis

**Affiliations:** Department of Biochemistry, University of Oxford, Oxford, United Kingdom; Washington University School of Medicine, UNITED STATES

## Abstract

*Plasmodium falciparum* Erythrocyte Membrane Protein 1 (*Pf*EMP1) and Knob-associated Histidine-rich Protein (KAHRP) are directly linked to malaria pathology. *Pf*EMP1 and KAHRP cluster on protrusions (knobs) on the *P*. *falciparum*-infected erythrocyte surface and enable pathogenic cytoadherence of infected erythrocytes to the host microvasculature, leading to restricted blood flow, oxygen deprivation and damage of tissues. Here we characterize the interactions of *Pf*EMP1 and KAHRP with host erythrocyte spectrin using biophysical, structural and computational approaches. These interactions assist knob formation and, thus, promote cytoadherence. We show that the folded core of the *Pf*EMP1 cytosolic domain interacts broadly with erythrocyte spectrin but shows weak, residue-specific preference for domain 17 of α spectrin, which is proximal to the erythrocyte cytoskeletal junction. In contrast, a protein sequence repeat region in KAHRP preferentially associates with domains 10–14 of β spectrin, proximal to the spectrin–ankyrin complex. Structural models of *Pf*EMP1 and KAHRP with spectrin combined with previous microscopy and protein interaction data suggest a model for knob architecture.

## Author summary

Formation of cytoadherent knobs on the surface of *P*. *falciparum* infected erythrocytes correlates with malaria pathology. Two parasite proteins central for knob formation and cytoadherence, KAHRP and *Pf*EMP1, have previously been shown to bind the erythrocyte cytoskeleton. Both KAHRP and *Pf*EMP1 include large segments of protein disorder, which have previously hampered their analysis. In this study we use biophysics and structural biology tools to analyze the interactions between these proteins and host spectrin. We devise a novel computational tool to help us towards this goal that may be broadly applicable to characterizing other complexes of widespread, disordered *Plasmodial* proteins and host components. We derive atomistic models of KAHRP–spectrin and *Pf*EMP1 –spectrin complexes, and integrate these into an emerging model of knob architecture.

## Introduction

Malaria remains one of the most lethal global diseases, causing an estimated 429,000 deaths in 2016 [1]. The majority of these deaths are attributed to infections by the *Plasmodium falciparum* parasite (reviewed in [2]). Compared to other human-infective *Plasmodia*, *P*. *falciparum* is distinguished by an extended set of proteins exported to erythrocytes it invades during the malaria blood stage [3–5]; these proteins remodel the host cell to assure ion homeostasis and increased nutrient uptake, and alter the host cell membrane structure and rigidity (reviewed in [6–8]). Particularly relevant to malaria pathology is the formation of protrusions on the *P*. *falciparum*-infected erythrocyte surface, known as knobs [9], that allow infected cells to adhere to uninfected erythrocytes and the microvascular endothelium. Cytoadherence of infected erythrocytes increases malaria severity by removing infected cells from circulation thereby allowing them to avoid splenic passage and clearance (reviewed in [10]). Further, accumulation of infected erythrocytes in the microvasculature disrupts blood flow, causes inflammation, and leads to oxygen deprivation in tissues and organ damage (reviewed in [2]). Thus, understanding the molecular mechanisms supporting knob formation in infected erythrocytes holds the potential of alleviating malaria severity by disabling parasite-induced cytoadherence.

Two key parasite factors for cytoadherence are the *Pf*EMP1 family [11–13] and KAHRP [14, 15]. *Pf*EMP1 members are the main protein adhesins presented on the surface of infected erythrocytes, where they cluster in knobs [16] and mediate direct interactions with human cell receptors (reviewed in [17]). KAHRP is essential for knob formation [18, 19], and knob-less parasitized cells lacking KAHRP lose the ability to cytoadhere under physiological blood flow conditions even though *Pf*EMP1 is still present at their surface [18, 20]. Both the *Pf*EMP1 family and KAHRP are unique to *P*. *falciparum*; thus, they are key members of the molecular arsenal responsible for severe malaria by this parasite (reviewed in [10]).

Knob density on the infected erythrocyte surface varies amongst parasite isolates and during intra-erythrocytic parasite development [21]; however, at high density knobs are spaced in regular intervals proportional to the extended length of host spectrin tetramers [19, 21, 22]. This correlation and the need to mechanically anchor adhesion molecules to cells in order to resist blood flow forces suggest the presence of links between knob components and the erythrocyte cytoskeleton. Pull-down assays *in vitro* and from parasitized cells support the presence of interactions between the *Pf*EMP1 cytosolic (intra-erythrocytic) domain and the cytoskeletal junction [23], and between KAHRP, spectrin and ankyrin [24–27]. Further, computational simulations of infected erythrocytes that assumed linkages between knobs and the cytoskeleton showed excellent agreement with experimental cell rigidity data [28], thereby supporting the notion that knob and cytoskeletal components interact.

Despite their importance in knob formation and, thus, disease pathology, none of the cytoskeletal connections formed by knob components have been studied in structural detail. Importantly, a coherent knob model integrating the different proposed interactions in a mechanistic picture is also lacking. Here we present a complementary biophysical, structural and computational analysis of KAHRP and *Pf*EMP1 interactions with erythrocyte spectrin, leading to atomistic models of how these two parasite proteins associate with the host cytoskeleton. We note the preference of both KAHRP and *Pf*EMP1 to associate adjacent to existing cytoskeletal complexes, and propose a model for knob architecture.

## Results

### The KAHRP 5´ repeat binds spectrin proximal to the ankyrin interaction site

**Fig 1:**
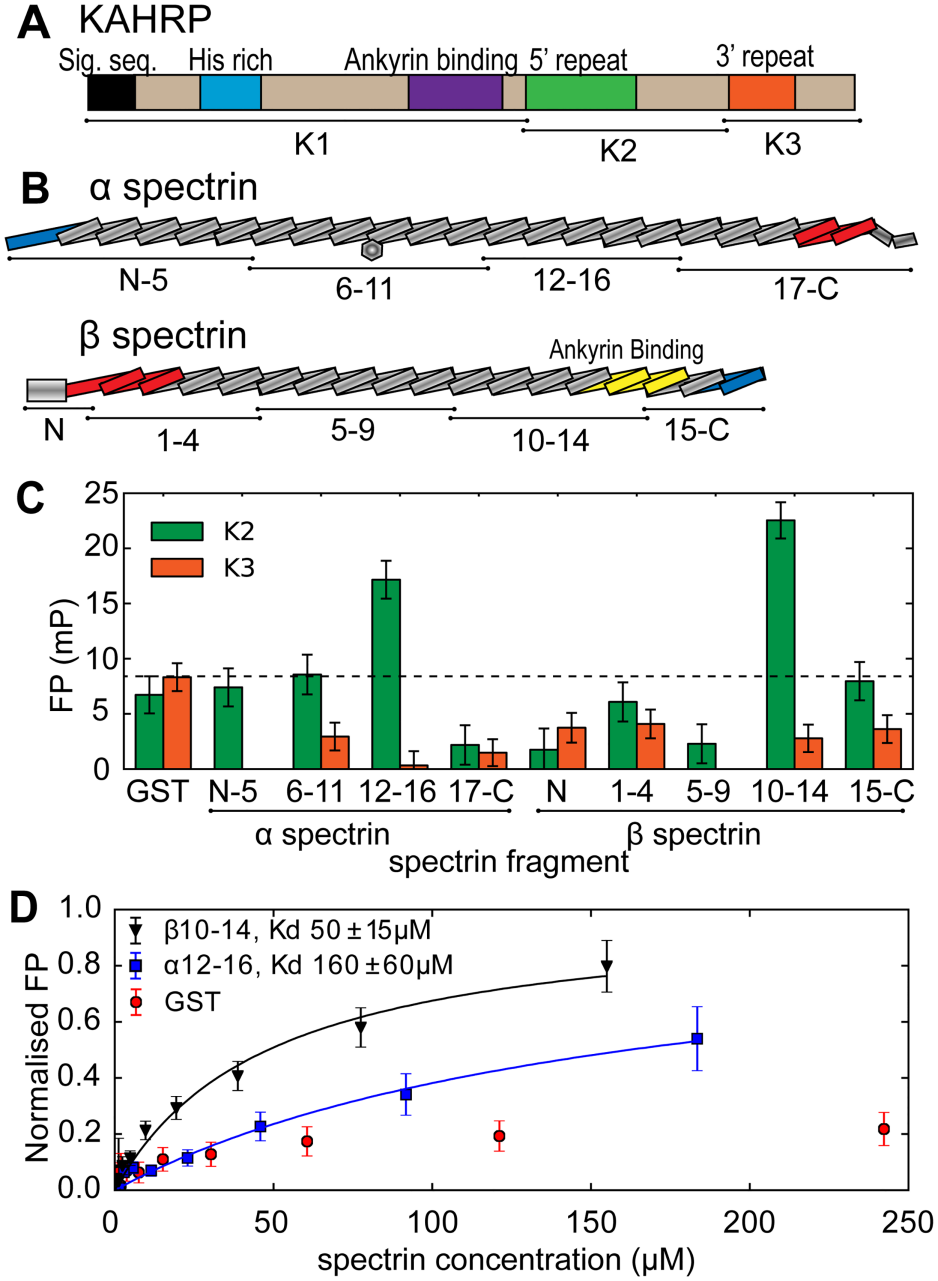
KAHRP associates with spectrin. (A,B) Schematic representations of KAHRP (A) and the spectrin α and β chains (B). KAHRP and spectrin fragments used in this study are denoted. The spectrin hetero-dimerization region is indicated in blue and the tetramerization region in red. (C) FP binding assay of KAHRP showing polarization differences (mP) upon incubation of fluoresceine-labeled K2 or K3 fragments with 50 μM of unlabeled spectrin constructs. Higher polarization differences correspond to slower tumbling rates of labeled KAHRP due to complex formation. The dotted line indicates polarization differences upon incubation with glutathione-S-transferase (GST, negative control). (D) FP titration of labeled K2 with unlabeled β10–14 and α12–16. Solid lines are fits of single site binding models; FP values were normalized to predicted maxima from the fits. The calculated interaction affinities (K_d_) are shown. Panels C and D show representative data from two independent experiments, while error bars indicate one standard deviation derived from four technical repeats.

KAHRP is a ~650 amino acid protein predicted to be highly disordered (Fig 1A and S1 Fig). The N-terminal half, referred to as K1, includes the eponymous histidine-rich region and fragments therein have been demonstrated to associate with the erythrocyte membrane [19] and ankyrin [25, 27]. The C-terminal half of KAHRP, divided into K2 and K3 segments, comprises two amino acid sequence repeat elements (5´ and 3´). The KAHRP C-terminal half and fragments therein also localize to the erythrocyte periphery [29] and associate with spectrin [24, 26], a multi-domain protein primarily composed of triple helical bundles (reviewed in [30]. We aimed to localize the interaction of KAHRP with spectrin, which is the most abundant cytoskeletal component. To that end we incubated fluorescently labeled recombinant K2 and K3 with fixed concentrations of recombinant spectrin constructs spanning both spectrin α and β chains (Fig 1B, a complete list of protein constructs is shown in Table 1), and measured increases in fluorescence polarization (FP) that result from slower tumbling of the labeled protein upon complex formation (Fig 1C). Two spectrin constructs spanning α chain domains 12–16 (henceforth α12–16) and β chain domains 10–14 (β10–14) produced significant increases of polarization with K2 indicative of binding. In contrast, we observed no spectrin association with K3. Titrations of labeled K2 with unlabeled α12–16 and β10–14 monitored by FP provided estimates for the interaction strength, K_d_, of 160±60 μM and 50±15 μM, respectively (Fig 1D).

**Table 1:** List of recombinant protein constructs.

Protein name & UniProt number	Construct name	Amino acid boundaries
*Spectrin domains*		
α spectrin, P02549	αN-5	1–585
α spectrin, P02549	α6–11	576–1185
α spectrin, P02549	α12–16	1174–1716
α spectrin, P02549	α17-C	1704–2395
α spectrin, P02549	α4	365–477
α spectrin, P02549	α16–19	1599–2036
α spectrin, P02549	α17–19	1704–2036
α spectrin, P02549	α18–19	1810–2036
α spectrin, P02549	α19–21	1923–2261
α spectrin, P02549	α16–17	1599–1828
α spectrin, P02549	α17	1704–1828
α spectrin, P02549	α19	1923–2036
β spectrin, P11277	βN	1–287
β spectrin, P11277	β1–4	295–747
β spectrin, P11277	β5–9	741–1278
β spectrin, P11277	β10–14	1272–1804
β spectrin, P11277	β15-C	1795–2137
β spectrin, P11277	β10–13	1272–1690
β spectrin, P11277	β11–14	1379–1804
β spectrin, P11277	β12–15	1475–1907
β spectrin, P11277	β9–11	1165–1482
β spectrin, P11277	β10–12	1272–1588
β spectrin, P11277	β11–13	1379–1690
β spectrin, P11277	β12–14	1585–1907
*PfEMP1 ATS fragments*^a^		
PF08_0141	ATS (for fluorescence)	1–393, Q150C^b^
PF08_0141, Q8IAK2	ATS-N	1–108
PF08_0141, Q8IAK2	ATS-M	137–244
PF08_0141, Q8IAK2	ATS-Core	109–136 and 245–292
PF08_0141, Q8IAK2	ATS-C	293–393
PF08_0141, Q8IAK2	ATS-NCore	1–136 and 245–292
PFF0845c, C6KT15	ATS (for fluorescence)	1–348, H159C^b^
PFF0845c, C6KT15	ATS-Core PFF0845c	118–145 and 264–313
PFB_1055c, O96296	ATS (for fluorescence)	1–410, Q161C^b^
PFC1120c, O97312	ATS (for fluorescence)	1–428, Q182C^b^
PFF0010w, C6KSK8	ATS (for fluorescence)	1–402, G156C^b^
PF08_0103, Q8IAS7	ATS (for fluorescence)	1–418, H154C^b^
*KAHRP fragments*		
KAHRP, Q9TY99	K1	38–362
KAHRP, Q9TY99	K2	356–533
KAHRP, Q9TY99	K3	533–654
KAHRP, Q9TY99	K1D	282–346
KAHRP, Q9TY99	K2-2	407–533
KAHRP, Q9TY99	K2-3	424–533
KAHRP, Q9TY99	K2-4	356–470
KAHRP, Q9TY99	K2-5	356–449
KAHRP, Q9TY99	K2-6	356–419
*KAHRP 5´-repeat scrambled sequences*		
Scramble 1	CGGSDNSEKNKKEHKKEKSAHEKEKDKKAEHKKDKDGSKKSSEKKKNSTKEHESSEHNHSEHKKSDNHKKKKENKEDDSEKEKNKKSDVVSDNKKTHKKGGKKKSKPKGNKSVHD	
Scramble 2	CEKSDDKKESKSKHDGSVKKKSEEKKKDNESSTKSDGKNKKNKKHNEHESKHEHKGEGKKKEHGSNEDHKKKNDNEVKGKHKKSPKKKAETKKNSSHDSDKKEKDSSKKVHNEAK	
Scramble 3	CHDNKGKKSHDSSGDKKHKNKKSKKKKESPTVDKKEKHEVKKKHKNKKDDVHSEEASKEENKKTEKGKNEKESNAKKSKNSHHDSKKGKEHEGSSKNKDHDKKEKNSDESKKGES	
Scramble 4	CESNKKNGHEKGGKKSESKHKKKDHAEKEPKNKKGAHHDDSDKSKTKHNSESDEKVHKSESKEKSKDKVGNKGKEKKVSNSEKKDKNKKDHSHSKETKEKDEKDKKHKSSNKENK	

^a^ ATS numbering starts at the first of three lysine residues following the *Pf*EMP1 transmembrane helix [31].

^b^ Single residue substitutions to cysteine were introduced to facilitate specific labeling with fluorescein-5-maleimide.

**Fig 2:**
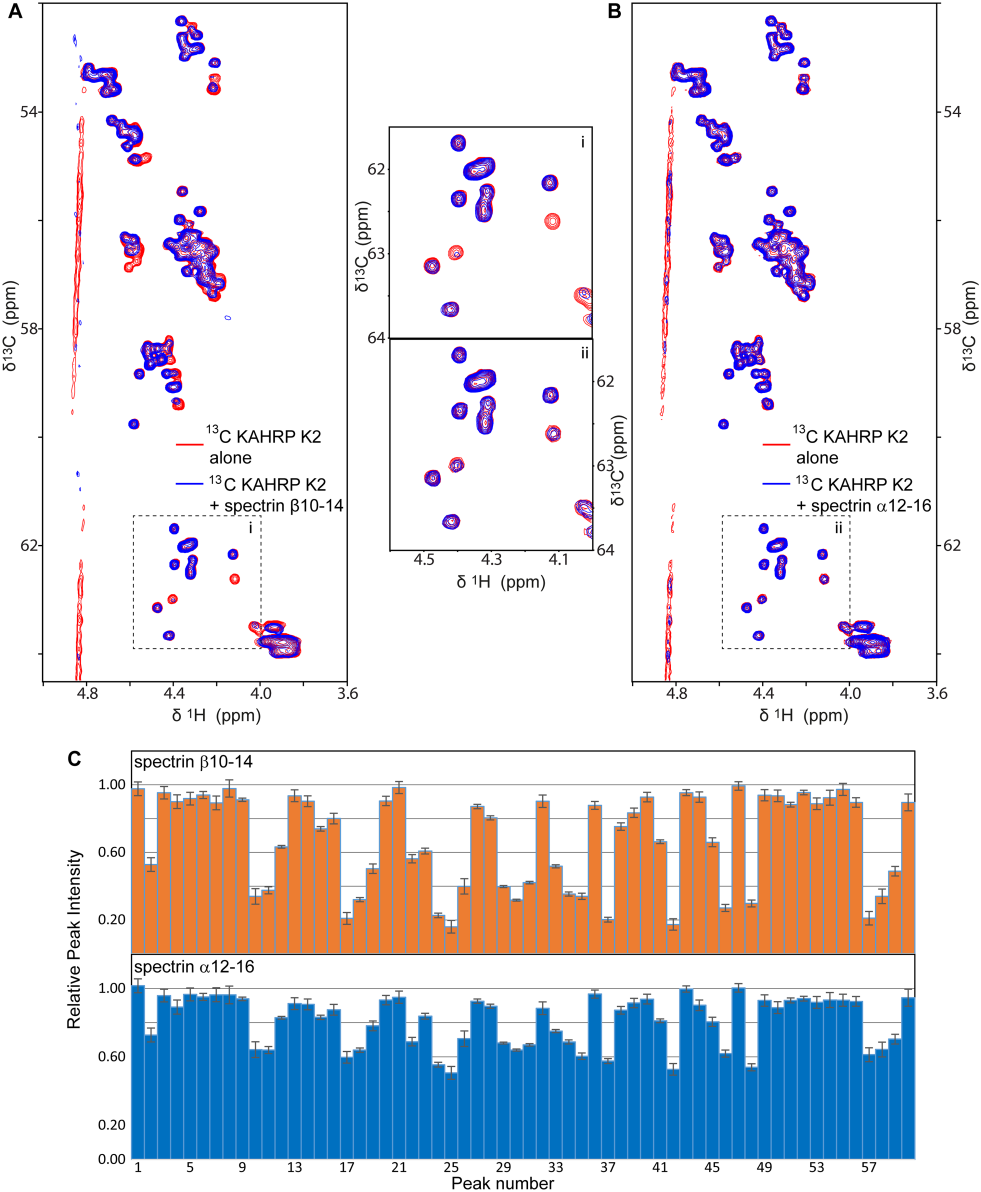
NMR-monitored titrations of KAHRP with spectrin. (A,B) Overlays of regions from ^13^C-HSQC NMR spectra of 50 μM of ^13^C-labeled KAHRP K2 alone (red) and in the presence (blue) of 150 μM unlabeled spectrin β10–14 (A) or α12–16 (B). Spectra were recorded at 10°C. Panels i and ii in the middle present magnifications of areas from the panels A and B, respectively. (C) The relative intensity of all discrete NMR peaks corresponding to C_α_ atoms of KAHRP K2 in the presence of β10–14 (top) or α12–16 (bottom), derived from panels A and B, respectively. Reduction of peak intensities suggests association between proteins. Error bars correspond to one standard deviation and derive from the signal / noise ratio of spectra.

Our localization of the KAHRP–spectrin interaction matches that of Pei *et al*. [26] for KAHRP; however, this earlier study suggested that K2 binds to spectrin α chain domain 4 (α4), whereas our FP experiments indicated binding to spectrin β10–14 and, to a smaller extend, to spectrin α12–16 (Fig 1C). To resolve this ambiguity we tested for KAHRP–spectrin binding using an independent biophysical method, Nuclear Magnetic Resonance (NMR). NMR spectra derived using isotopically labeled samples are sensitive to the protein structure and the chemical environment, both of which change upon direct protein–protein binding. Furthermore, NMR allow us to simultaneously observe signals from multiple different areas of a protein, as these give rise to distinct peaks in the NMR spectrum, thereby helping us to avoid false positive or negative results. We acquired NMR spectra of ^15^N-labeled α4 in the presence of unlabeled K2 and saw no differences in the positions or intensities of NMR peaks compared to spectra of labeled α4 alone, which suggests that K2 and α4 do not interact (S2 Fig). In contrast, NMR spectra of ^13^C-labeled K2 with unlabeled β10–14 showed reduction in intensity of 50% or more for approximately 40% of distinct NMR peaks compared to spectra of labeled K2 alone (Fig 2A and 2C), which is indicative of direct binding. Similar spectra of ^13^C-labeled K2 with unlabeled α12–16 with yielded smaller reductions in peak intensities, consistent with a weaker interaction between these components (Fig 2B and 2C). We conclude that K2 binds β10–14 with ~3-fold affinity preference compared to α12–16, and even higher specificity against other spectrin sub-fragments (Fig 1C and 1D). Notably, β10–14 is adjacent to the ankyrin binding site on spectrin domains β14–15 [32].

### KAHRP and spectrin interact over a broad epitope

The NMR spectra of K2 showed substantial overlap of peaks, in agreement with previous NMR studies of K2 fragments [33]. Notably, we were able to resolve only 60 unique NMR peaks corresponding to K2 ^13^Cα atoms out of 178 possible peaks in total (Fig 2). This prevented us from assigning specific NMR peaks to individual KAHRP amino acids, which would have enabled us to narrow down the spectrin interaction epitope on K2. Thus, to further localize the KAHRP–spectrin interaction we instead performed FP titrations of labeled K2 sub-fragments with β10–14, and similar titrations of labeled K2 with overlapping spectrin sub-fragments. We found that KAHRP fragments composed of just the 5´ repeat region retained full β10–14 binding affinity (S3A and S3C Fig); in contrast, KAHRP truncations that removed elements of the 5´ repeat reduced β10–14 binding in a manner proportional to the number of repeat elements eliminated (S3A, S3C and S3D Fig). Deletion of one or more β spectrin domains also resulted in step-wise reduction of K2 affinity (S3B and S3C Fig). Considered together these titrations did not support the presence of a narrow, highly localized interaction epitope between KAHRP K2 and spectrin β10–14 but, rather, indicated a broad association over many spectrin domains and KAHRP 5´ repeat elements.

### Visualizing the KAHRP–spectrin complex

Despite repeated attempts we were unable to obtain diffracting crystals of the KAHRP–spectrin complex. In the absence of crystallographic data or well dispersed NMR spectra, we set out to model this complex in order to understand what drives the preferential association of KAHRP 5´ repeat with β10–14. Although a number of computational tools allow docking of protein fragments to folded domains [34–36], these are typically limited to relatively small disordered peptides (<30 amino acids, compared to 115 amino acids of the KAHRP 5´ repeat) and most require some initial knowledge of the complex structure; thus, on both counts these tools were not suitable for modeling the KAHRP–spectrin complex. We noted that both β10–14 and the KAHRP 5´ repeat are highly enriched in ionic amino acids and carry opposite charges (negative charge, calculated pI of 4.9 for β10–14; positive charge, pI of 9.65 for the KAHRP 5´ repeat; S3D and S3E Fig), suggesting that their binding is driven by electrostatic interactions. Indeed, FP titrations of labeled K2 with unlabeled β10–14 showed reduction of binding affinity as a function of increased ionic strength (S3F Fig). Thus, we examined whether electrostatic complementation might provide an initial basis for modeling the β10–14 –KAHRP 5´ repeat complex.

**Fig 3:**
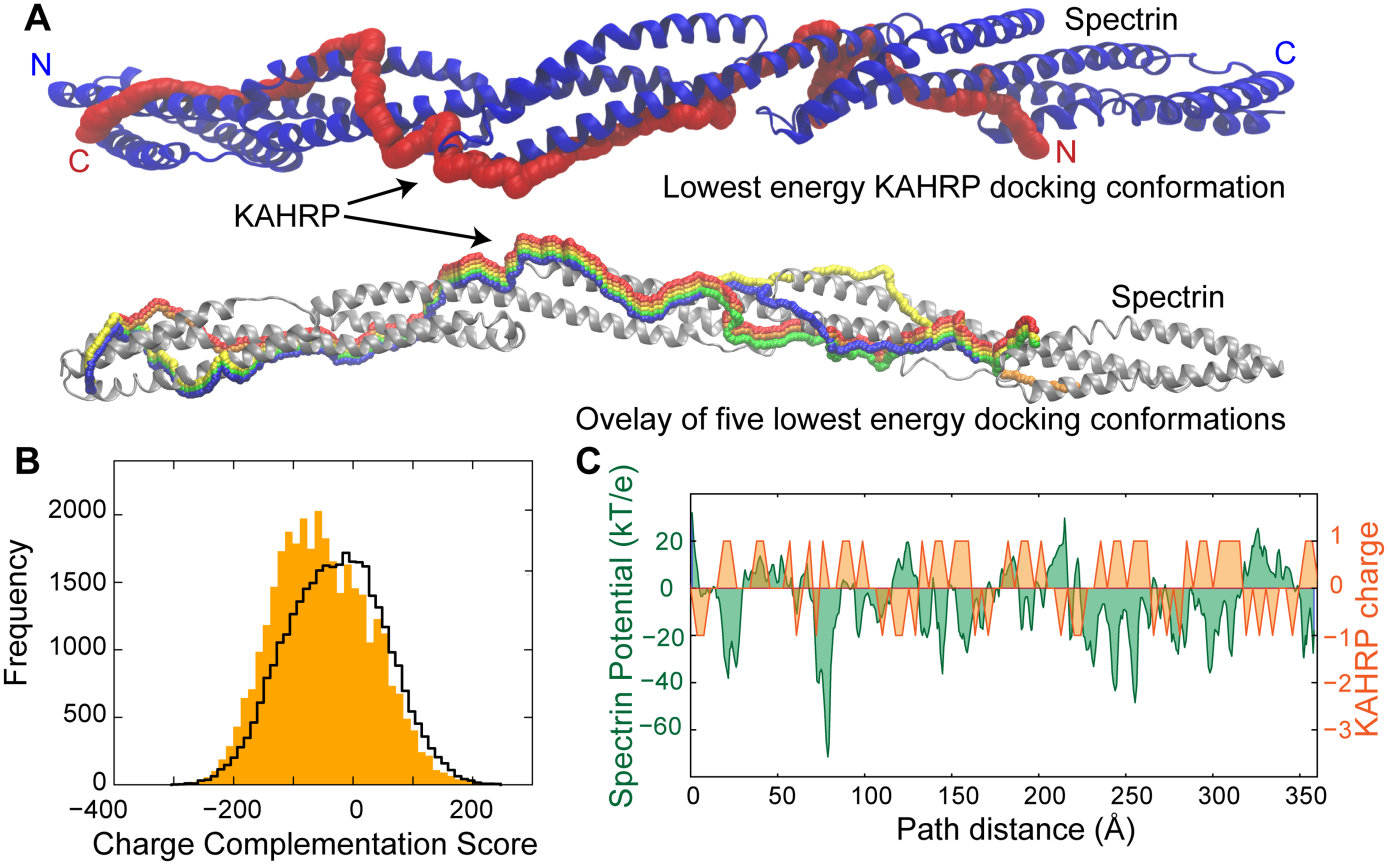
Electrostatic docking of KAHRP and spectrin. (A) Schematic representation of KAHRP 5´ repeat conformations upon binding to β10–14. The spectrin fragment is shown in cartoon representation, and its N- and C-terminus are denoted. The red KAHRP conformation (path, top panel) achieved the lowest (most favorable) docking energy (score). An overlay of the five lowest energy KAHRP paths is shown at the bottom panel. (B) Histogram of the docking (charge complementation) score calculated for all putative KAHRP 5´ repeat paths along β10–14. Filled bars correspond to docking scores calculated for antiparallel orientations of KAHRP with spectrin; a black line denotes docking scores from parallel orientations. (C) Graph of the electrostatic potential of the spectrin β10–14 surface along the length of the lowest scoring path (kT/e, green), overlaid with the formal charge of the KAHRP 5´ repeat (orange). The relative KAHRP and spectrin positions along the docking path are denoted using a distance calculated from the β10–14 N-terminus.

We developed a novel computational docking tool that attempts to predict the binding conformation between a folded protein (in this case, β10–14) and a disordered component (the KAHRP 5´ repeat) on the basis of electrostatic interactions (see Materials and methods for detailed methodology, and S4 and S5 Figs for benchmarking of the new tool). Using this bespoke tool we identified a number of paths on the β10–14 surface that displayed remarkable charge complementation to the KAHRP 5´ repeat (Fig 3) and, thus, had low (favorable) interaction scores. We noted that the five best scoring paths tracked a broadly similar trajectory on β10–14 (Fig 3A) and that antiparallel KAHRP–spectrin conformations were generally favored (Fig 3B). To further refine the docked β10–14 –KAHRP 5´ repeat complex we performed triplicate atomistic molecular dynamics (MD) simulations starting from the most favorable docking conformation (Fig 3A). Similar to MD simulations of complexes determined by high-resolution methods, our simulations converged rapidly (S6A and S6B Fig) to models showing interactions between charged KAHRP residues and complementary charged clusters of spectrin (S6C Fig). Interestingly, the MD simulations also showed evidence of a dynamic behavior in the KAHRP–spectrin complex as demonstrated by small changes in the binding conformation (S6D Fig). We surmise that realistic models of the β10–14 –KAHRP 5´ repeat complex can be generated by considering the charge complementarity of these two proteins.

### The KAHRP–spectrin complex is partly sequence specific

We earlier observed that the KAHRP 5´ repeat shows at least 3-fold affinity preference for β10–14 compared to other spectrin fragments (Fig 1C and 1D), which may arise as a result of fine electrostatic complementation between these two proteins (Fig 3C). Such fine complementation could provide the molecular basis for sequence specificity in this interaction. In order to test the specificity of β10–14 –KAHRP 5´ repeat binding we produced four recombinant peptides with amino acid content equivalent to the KAHRP 5´ repeat but randomly scrambled sequences (Table 1 and S7A Fig). FP titrations of labeled scrambled peptides with β10–14 revealed up to 5-fold reduction in affinity compared to the canonical KAHRP 5´ repeat (S7C Fig). Consistent with this reduction in affinity, docking of the four scrambled peptides to β10–14 using the electrostatic complementation tool above yielded less favorable scores compared to the canonical KAHRP 5´ repeat (S7B and S7C Fig). We conclude that the binding between β10–14 and the KAHRP 5´ repeat is enhanced 2- to 5-fold by sequence specific interactions, which is comparable to the overall margin of specificity observed for KAHRP binding to spectrin fragments in general. However, a broader non-specific electrostatic interaction between the negatively charged spectrin and positively charged KAHRP is also present.

### The *Pf*EMP1 cytoplasmic domain binds spectrin proximal to the junctional complex

**Fig 4:**
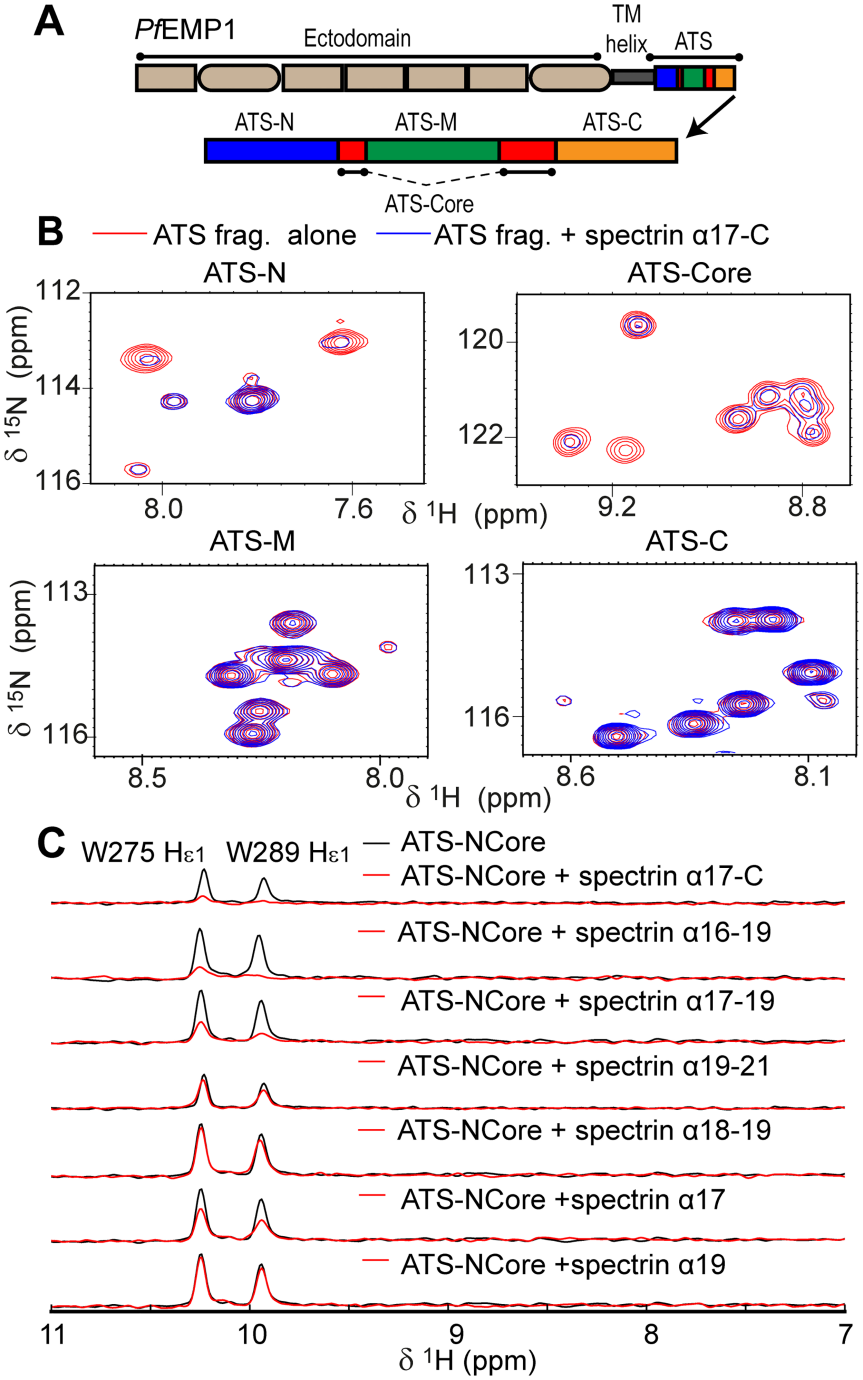
ATS interacts with spectrin. (A) Schematic representation of *Pf*EMP1 and its intracellular ATS domain. The ATS fragments are denoted; the ATS-Core is composed of two discontinuous sections that fold into a single globular unit. (B) Details from NMR ^15^N-HSQC spectra (S9 Fig) of 100 μM ^15^N-labeled ATS fragments in the absence (red) and presence (blue) of equimolar unlabeled α17-C. (C) 1D traces of NMR spectra showing ATS-Core peaks alone (black) or in the presence (red) of α17-C sub-fragments. Traces derive from ^15^N-HSQC spectra of 100 μM ^15^N-labeled ATS-NCore with two-fold excess of unlabeled spectrin sub-fragments. The ATS atoms giving rise to these NMR peaks are indicated. Reduction of peak intensities suggests association between proteins.

Similar to KAHRP, the cytoplasmic domain of *Pf*EMP1, known as Acidic Terminal Segment (ATS), is primarily disordered [31]. ATS comprises a small folded core (ATS-Core; Fig 4A) and flexible segments at its N-terminus (ATS-N), middle (ATS-M) and C-terminus (ATS-C). The ATS architecture is conserved across *Pf*EMP1 variants [31]. As ATS binds components of the erythrocyte spectrin–actin–band 4.1 complex [23] we examined the ability of fluorescently labeled ATS from *Pf*EMP1 variant PF08_0141 to bind spectrin sub-fragments (S8A Fig). ATS displayed weak affinities for most spectrin sub-fragments in these assays; however, it bound with ~2-fold preference to an α spectrin construct spanning domains 17 to the protein C-terminus (α17-C, K_d_ = 59±6 μM).

To independently validate the ATS–spectrin association, and to further localize the interaction epitope, we performed NMR experiments where ^15^N-labeled ATS-N, ATS-Core, ATS-M and ATS-C were titrated with unlabeled α17-C (Fig 4B and S9 Fig). We observed no evidence for an α17-C interaction with ATS-M or ATS-C (S9E and S9F Fig), whereas NMR spectra of ATS-N and, especially, ATS-Core showed perturbations in peak positions indicative of binding (S9A–S9D Fig). Similar NMR assays and FP titrations with overlapped α17-C sub-fragments uniquely localized the ATS interaction on domain α17 (Fig 4C and S8B Fig). To test whether the ATS–α17 interaction is conserved among *Pf*EMP1 variants we produced a further five fluorescently labeled full-length ATS domains whose divergent sequences are representative of the *Pf*EMP1 family in general [31]. All ATS variants bound α17; however, the interaction affinities varied between 24 μM and 200 μM (S8C Fig). Thus, we conclude that *Pf*EMP1 ATS feature a conserved association with spectrin that shows weak preference for α17, which is proximal to the cytoskeletal junctional complex.

### The ATS–spectrin binding is sequence specific

**Fig 5:**
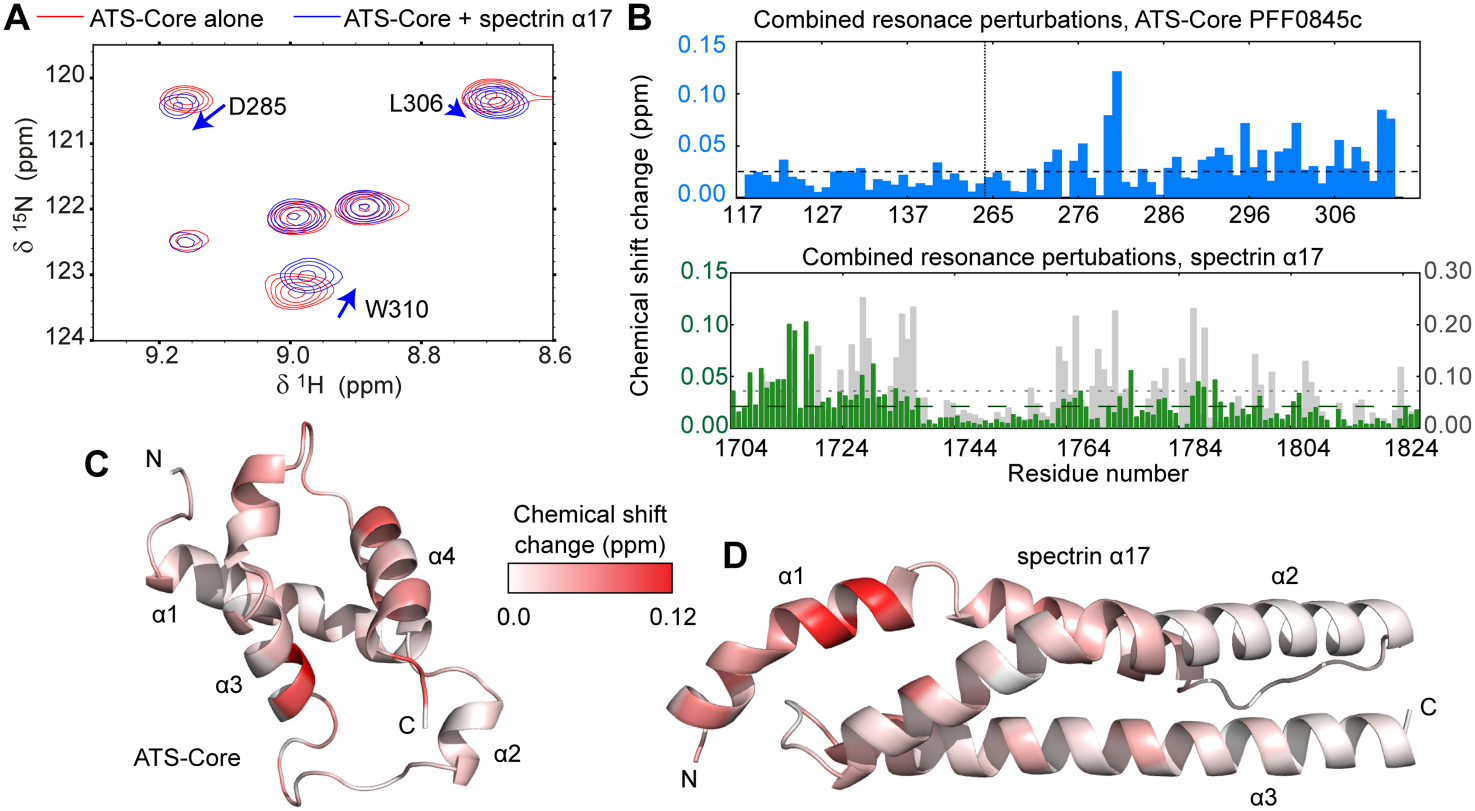
Mapping the ATS-Core–spectrin α17 binding on the domain structures. (A) Detail from NMR ^15^N-HSQC spectra of 50 μM ^15^N-labeled ATS-Core PFF0845c alone (red) or with two-fold excess of unlabeled α17 (blue). Spectra were recorded at 300 mM NaCl and 40°C. (B) Combined perturbations of ^1^H, ^15^N and ^13^C´ NMR peak positions upon ATS-Core–spectrin α17 binding under the conditions of (A), as function of ATS-Core PFF0845c (blue) and spectrin α17 (green) amino acids. Combined perturbations of ^1^H and ^15^N NMR peak positions for α17 upon ATS-Core binding at 50 mM NaCl and 25°C are also shown (grey); NMR peak perturbations are broadly similar under both experimental conditions. Mean perturbations in NMR peak positions are shown as dashed lines. The position at which *Pf*EMP1 segments are joined to form the ATS-Core is indicated by a dashed line in the top graph. (C,D) Visualization of NMR peak position perturbations upon ATS-Core–α17 binding on (C) the ATS-Core and (D) the α17 structure. The color gradient indicates the magnitude of perturbations observed; thus, it is proportional to structural changes upon complex formation allowing the visualization of the direct interaction interface. The ATS-Core PFF0845c structure was derived by homology modeling using the ATS-Core PF08_0141 structure [31] as template. The two constructs feature 70% sequence identity and 92% sequence similarity. The α17 structure derives from a 1.54 Å resolution crystallographic model of spectrin repeats α16–17 (See S10 Fig and Table 2 for an analysis of α16–17 structure).

**Fig 6:**
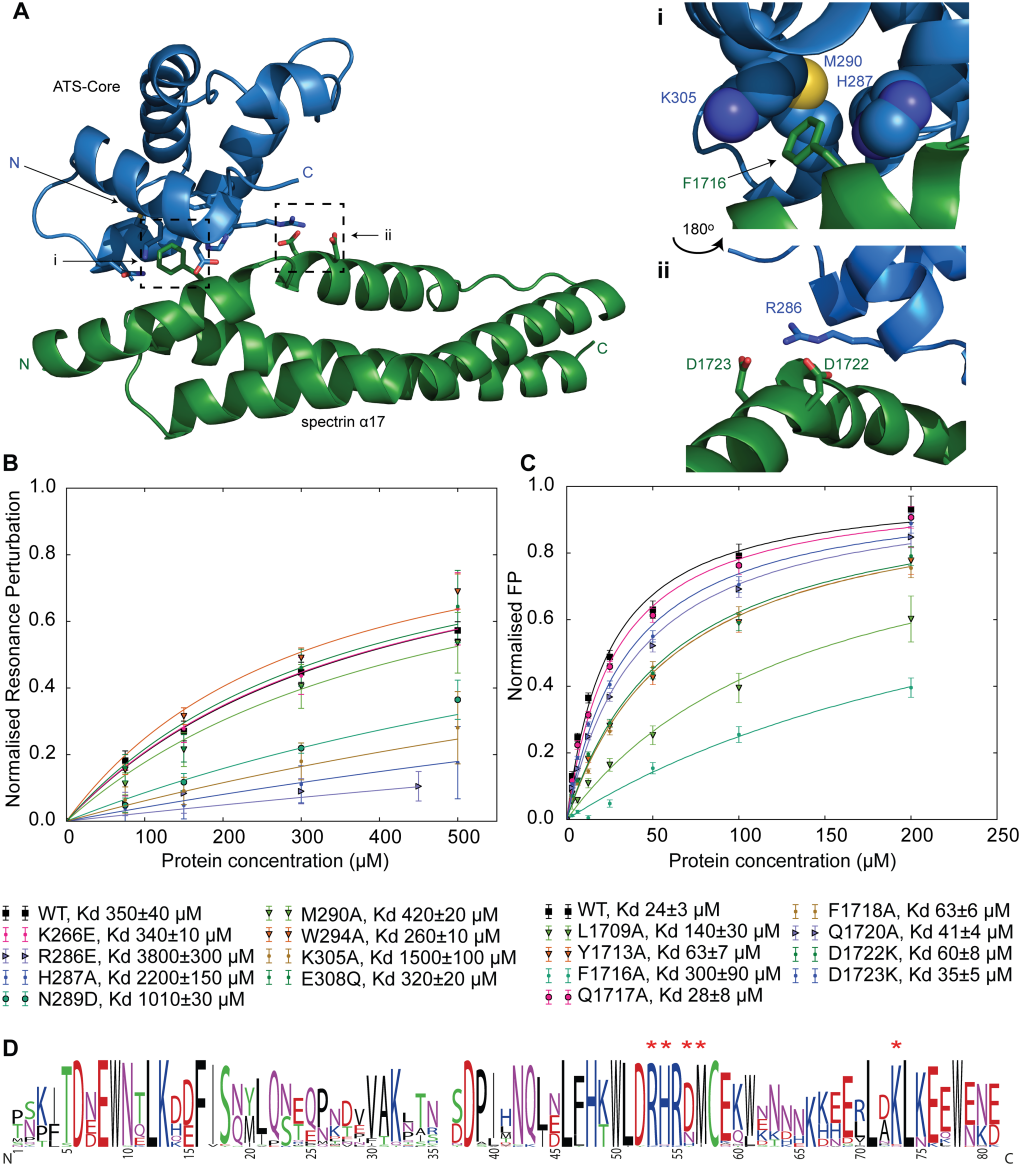
Model of the ATS-Core–α17 complex. (A) Overview of the ATS-Core–α17 complex following refinement by MD simulations. The protein N- and C-termini, and interfacial residues are indicated. Right panels correspond to hydrophobic (i) and electrostatic and hydrogen bonding (ii) features observed in the complex structure. Panel ii is rotated 180° compared to panel i. (B) NMR-monitored titrations of 50 μM ^15^N-labeled α17 with unlabeled ATS-Core PFF0845c variants that carry amino acid substitutions at the binding interface. Experiments were performed at 50 mM NaCl and 25°C. Each data point represents the average fractional perturbation in the position of the eight most affected α17 NMR peaks. Error bars indicate one standard deviation and derive from variances in fractional changes across the eight resonances. Solid lines represent fits to single site binding models. Interaction affinities are indicated. (C) FP titrations of fluoresceine-labeled ATS PFF0845c with α17 variants carrying amino acid substitutions at the binding interface. Error bars indicate one standard deviation and derive from three replicates each with four technical repeats. Solid lines represent fits to single site binding models. (D) Consensus sequence of ATS-Core domains from *Pf*EMP1 variants in the *P*. *falciparum* 3D7 strain. Red asterisks above residues indicate ATS-Core amino acids whose substitution affects α17 binding affinity. Residue numbers correspond to the sequence of the ATS-Core structured domain after removal of loop insertions [31].

As ATS variant PFF0845c binds α17 with substantially higher affinity than other members of this family (S8C Fig), we characterized that complex aiming to identify specific amino acids at the binding interface (Fig 5). Comparison of α17 affinity to full-length ATS or ATS-Core suggested that the folded ATS core comprises most of the interaction interface (~75% of the binding energy based on measured affinities; Fig 6B and 6C). We performed NMR titrations with ATS-Core PFF0845c and α17 (Fig 5A) and mapped the residues most significantly affected by complex formation as revealed by perturbations in NMR peak positions (Fig 5B). Affected residues primarily cluster on α-helix 1 of α17 and on the C-terminal helical hairpin of the ATS-Core (Fig 5C and 5D). Docking the ATS-Core and α17 structures using the NMR peak position perturbations as distance restraints resulted in the prediction of two possible complex conformations that are related by an approximately 170° rotation of the ATS-Core (S11A and S11B Fig).

To distinguish between these two possibilities we refined both complex conformations by triplicate MD simulations (S11C and S11D Fig) and observed that one conformation (complex 1) remained relatively unaltered, as the complex components deviated little from their starting positions. In contrast, the second complex conformation (complex 2) was destabilized in the MD simulations and occasionally completely disrupted, which suggested that this conformation is likely incorrect. Further, we noted that complex 1 showed a number of hydrophobic, ionic and hydrogen bond interactions similar to those found in high-resolution structures of protein complexes, such as the insertion of α17 F1716 into an ATS-Core hydrophobic cavity formed by residues H287, M290 and K305, and the interaction of R286 of ATS-Core with D1722 / D1723 of α17 (Fig 6A). In contrast, complex 2 lacked these features.

To validate the ATS-Core–α17 complex we substituted residues at the binding interface and quantified the effect of these substitutions on interaction affinity (Fig 6B and 6C) using NMR and FP titrations. We observed that single substitutions of specific ATS-Core and α17 amino acids reduced affinity by up to 10-fold. Furthermore, we noted that ATS-Core residues at the α17 binding interface are conserved or conservatively substituted across all *Pf*EMP1 ATS-Core variant (Fig 6D). Considering these results in combination, we surmise that ATS-Core forms a specific and conserved complex with spectrin α17, which is sensitive to disruption by mutagenesis.

## Discussion

The importance in malaria pathology of knob protrusions on the surface of *P*. *falciparum*- has been well established for over thirty years (e.g. [9, 37]), yet our understanding of the protein interactions underpinning their formation remains incomplete. Such understanding could provide crucial insight on the evolution, assembly and mechanistic characteristics of knobs, and possibly lead to avenues for knob disruption and reduction of *P*. *falciparum*-infected erythrocyte cytoadherence. Recently, we and co-workers provided the first structural details on knob components and complexes, including the structure of the *Pf*EMP1 intracellular domain ATS [31], the structure of a *Pf*EMP1- and cytoskeleton-associated parasite PHIST protein [38, 39], and the first glimpse of knob architecture by electron tomography, which revealed the formation of a spiral scaffold underneath knobs by an unknown protein component [40]. Here, we complement this picture through the detailed analysis of interactions between two crucial knob components, *Pf*EMP1 and KAHRP, with the major cytoskeletal component in erythrocytes, spectrin.

Our analysis suggests that the KAHRP 5´ repeat preferentially associates with a specific segment of erythrocyte spectrin, β10–14 (Fig 1). Although the affinity of KAHRP 5´ repeat for β10–14 is relatively weak, it is comparable to the strength of intracellular interactions seen in other adhesion-related complexes, such as those in animal focal adhesion assemblies [41]. We combined a novel electrostatic docking tool, MD simulations and a battery of biophysical affinity measurements to characterize the complex between the KAHRP 5´ repeat and spectrin β10–14. We found that complex formation is driven by electrostatic interactions, which are individually weak, and that optimal affinity requires multiple KAHRP and spectrin repeats. Furthermore, our assays revealed that this complex is partly sequence-specific but also underpinned by a more general electrostatic attraction between KAHRP and spectrin.

**Fig 7:**
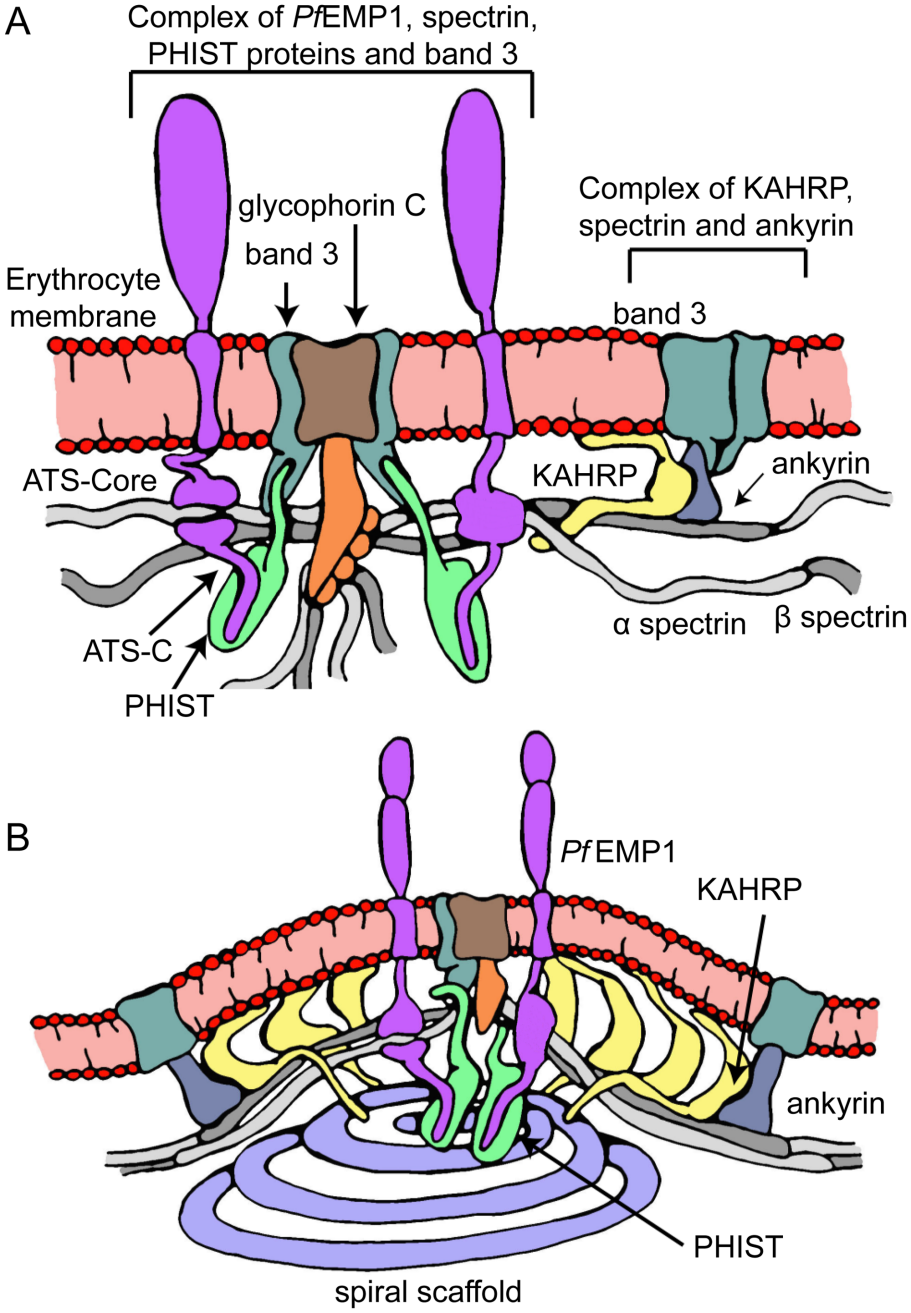
Model of knob formation structure. Panel (A) depicts an initial step in knob assembly when *Pf*EMP1 and KAHRP have localized close to the junctional and ankyrin complexes, respectively. PHIST proteins, such as PFE1605w [38, 39, 44], may further connect *Pf*EMP1 and the cytoskeleton. Panel (B) depicts a model of the final knob structure, characterized by high protein density as a result of KAHRP self-association [23] and the formation of spiral assemblies [40].

Previous work demonstrated the functional significance of the KAHRP 5´ repeat through deletions of this protein region in transgenic parasites, which disrupted canonical knob formation and resulted in reduced infected erythrocyte adhesion [19]. Our work shows that these earlier experiments would have disrupted the KAHRP–spectrin interaction, thereby suggesting that this interaction may be essential for robust cytoadherence. Furthermore, the preferred KAHRP interaction site on spectrin, β10–14, is proximal to the ankyrin interaction site at β14–15 [32], and earlier studies have indicated the existence of an ankyrin-binding epitope on KAHRP adjacent to the spectrin-binding 5´ repeat region (Fig 1A; [25, 27]). Thus, we postulate that in the context of the erythrocyte cytoskeleton a ternary KAHRP–spectrin–ankyrin complex may form (Fig 7A), which would serve to strengthen the KAHRP–cytoskeleton association and increase its specificity. In such a complex KAHRP would cross-link spectrin and ankyrin, an effect that may be partly responsible for the increase in cytoskeletal rigidity observed upon parasite infection of erythrocytes [19, 26].

As part of our KAHRP 5´ repeat–β10–14 analysis we developed a novel computational tool for docking flexible protein segments to structured components on the basis of electrostatic complementation. Compared to existing peptide docking tools [34–36] our approach does not require *a priori* knowledge of the relative position of the binding partners, and it is capable of handling very long peptides despite the increased conformational space available to such ligands. Our simplified methodology does not capture important binding details, such as hydrophobic interactions or hydrogen bonds; however, benchmarking of this electrostatic docking tool against known interaction affinities and a high-resolution complex structure revealed that it successfully reproduces experimental results. We anticipate that further elaboration of this tool, perhaps by taking advantage of MD simulations from approximate starting coordinates, may prove of general utility to studies of protein–peptide interactions. In particular, we note that highly-charged protein sequences are common in protozoan parasites, including *Plasmodium* [42, 43], and that such charged proteins are known to associate with the infected erythrocyte periphery [29].

Our analysis points to promiscuous spectrin binding by *Pf*EMP1 through its cytosolic domain, ATS (Fig 4), albeit with weak specificity for domain 17 of α spectrin, which is close to the cytoskeletal junctional complex (Fig 1). The ATS–α17 interaction affinity differs across *Pf*EMP1 variants, but in most cases it is comparable to that observed for KAHRP–spectrin binding. However, unlike the dynamic KAHRP–spectrin complex, ATS–α17 binding crucially depends on specific amino acids conserved among *Pf*EMP1 members. In the context of infected erythrocytes we anticipate that the *Pf*EMP1 –cytoskeleton binding will be strengthened by indirect interactions. Specifically, we showed earlier that *Pf*EMP1 ATS associates with band 3 protein via the parasite PHIST protein PFE1605w, also known as LyMP [44], which binds band 3 and ATS-C [38, 39, 44]. Interestingly, our results suggest that the *Pf*EMP1 variant exhibiting the strongest direct binding to spectrin, PFF0845c, has the weakest affinity for PFE1605w [39] and, hence, the weakest indirect association to the cytoskeleton. It is tempting to speculate that parasite evolution has sought to maintain the total strength of the *Pf*EMP1 –cytoskeleton connection, while flexibly utilizing two independent molecular mechanisms.

Crucially, both of these binding mechanisms target *Pf*EMP1 to the vicinity of the cytoskeletal junctional complex; thus, if acting together, these mechanisms have the potential to increase the strength and specificity of *Pf*EMP1 localization. The junctional complex is a privileged point in the cytoskeleton as it brings close together in space three to eight spectrin chains [45], actin, band 3 and band 4.1 (reviewed in [46]), therefore it has the potential to recruit multiple *Pf*EMP1 molecules and to drive their clustering on the erythrocyte surface independently of knob formation (Fig 7A), as observed experimentally in knob-less parasitized cells [20, 47]. Direct *Pf*EMP1 clustering via cytoskeletal interactions at the vicinity of the junctional complex may act synergistically with binding of the *Pf*EMP1 ectodomain by IgM and α2-macroglobulin, thereby contributing to strong cytoadherence [48–50].

Our work together with previous studies allows us to propose a model of knob architecture (Fig 7B). Under this model KAHRP is exported to the erythrocyte membrane where it binds the cytoskeleton at spectrin–ankyrin complexes, which are peripheral to cytoskeletal junctions. KAHRP binding leads to cytoskeletal rigidification [19, 26], and may be partly responsible for the increase spacing between integral membrane proteins at the knob apex and its periphery [40]. KAHRP may further self-associate as suggested by earlier studies [23] to form the electron-dense protein coat observed underneath knobs [15, 51]. Parallel to this process *Pf*EMP1 binds to PHIST members [38, 39] and clusters around the cytoskeletal junctional complex through direct binding to spectrin α17 and indirect interactions with band 3 [39]. Finally, a yet unknown parasite protein is recruited to the growing knob complex likely through interactions with knobs components and assembles into a spiral scaffold [40]. Thus, an outward membrane protrusion with apical adhesion molecules is formed, which allows strong infected erythrocyte engagement with other host cells.

## Materials and methods

### Protein purification

*P*. *falciparum* KAHRP (UniProt accession number Q9TY99) constructs, shown in Table 1, were cloned in a modified pET16b vector that includes an N-terminal His_10_-tag and a human rhinovirus (HRV) 3C protease cleavage site, and transformed into *Escherichia coli* strains BL21(DE3) CodonPlus-RP (Agilent Technologies, Stockport UK) or Rosetta2(DE3) (Novagen, Watford UK). Cells were grown at 37°C in Luria Bertani (LB) media or, for NMR usage, in M9 minimal media supplemented with ^15^N enriched NH_4_Cl and/or ^13^C enriched D-glucose. The growth temperature was reduced to 18°C at OD_600_ ~0.5, and protein expression was induced at OD_600_ ~0.6 with 500 μM final concentration of Isopropyl β-D-1-thiogalactopyranoside (IPTG, Generon, Maidenhead UK) for 16–18 hrs. Cells were harvested by centrifugation and resuspended in 50 mM NaH_2_PO_4_, 500 mM NaCl, 8 M Urea pH 7.8 buffer. Cells were lysed with sonication and lysates were clarified by centrifugation at 50,000 g prior to loading in Talon metal affinity columns (Clontech, Moutain View CA) equilibrated in lysis buffer. Proteins were eluted by lysis buffer supplemented with 500 mM imidazole, and extensively dialyzed against 500 mM NaCl, 50 mM NaH_2_PO_4_, 1 mM 1,4-dithiothreitol (DTT), 1 mM ethylenediaminetetraacetic acid (EDTA) pH 6.5 buffer. Cloning tags were removed by cleavage with recombinant HRV 3C protease. Proteins were dialyzed again 150 mM NaCl, 20 mM NaH_2_PO_4_, 1 mM DTT, 1 mM EDTA pH 6.5 buffer prior to ion exchange chromatography (SP-Sepharose media, GE Healthcare, Little Chalfont UK). Final purification was performed by size exclusion chromatography using Superdex 75 (GE Healthcare) media equilibrated in analysis buffer (20 mM NaH_2_PO_4_, 50 mM NaCl, 1 mM DTT pH 7) unless otherwise noted.

DNA fragments encoding scrambled KAHRP 5´ repeat sequences were made synthetically (IDT, Leuven Belgium) and cloned in a modified pEt16b as above. Scrambled peptides were produced recombinantly as described for KAHRP fragments above.

Human erythrocyte spectrin constructs (UniProt P02549 and P11277, Table 1) were cloned in modified pET16b (as above) or pET15b (N-terminal His_6_-tag, thrombin cleavage site) vectors and recombinately expressed in *E*. *coli* Rosetta2(DE3) in LB media for 4 hrs at 37°C following induction with 250 μM final concentration of ITPG. Cells were harvested by centrifugation and re-suspended in PBS (150 mM NaCl, 20 mM Na_2_HPO_4_ pH 7.4). Cells were lysed with sonication and lysates clarified by centrifugation and applied to PBS-equilibrated Talon metal affinity columns. Proteins were eluted using PBS supplemented with 500 mM imidazole, dialyzed against 50 mM Tris-Cl, 50 mM NaCl pH 7.5 buffer, and cloning tags were cleaved using HRV 3C or thrombin (Sigma Aldrich, Gillingham UK) proteases. Proteins were further purified by ion exchange chromatography (Q-Sepharose media, GE Healthcare) and size exclusion chromatography (Superdex 75 or 200 media, GE Healthcare) into analysis buffer unless otherwise noted.

Recombinant expression of *P*. *falciparum* Erythrocyte Membrane Protein 1 (*Pf*EMP1) Acidic Terminal Segment (ATS) variants and constructs was performed as described [31]. 5-Carboxyfluorescein (5-FAM) labeling of proteins for fluorescence assays used a previously established protocol [31]. 5-FAM labeling was performed in a site-specific manner using single cysteine residues introduced at the middle disordered segment of ATS variants [31] or at the protein N-terminus. Two pre-existing cysteine residues in the KAHRP K2 construct were substituted by alanine (C414A/C450A) using QuikChange mutagenesis (Agilent Technologies). Amino acid substitutions were introduced in ATS-Core and spectrin α17 constructs by QuikChange mutagenesis.

Proteins were concentrated by spin ultrafiltration, and concentrations estimated by UV absorption at 280 nm. Protein identity was confirmed by electrospray ionization mass spectrometry. All chemical reagents used were purchased from Sigma Aldrich unless otherwise noted.

### Fluorescence assays

Fluorescence polarization (FP) binding assays were performed at 20°C using a CLARIOStar fluorimeter (BMG Labtech, λ_ex_ = 485 nm, λ_em_ = 520 nm). 5-FAM-labeled proteins at 0.5 or 1 μM concentration in analysis buffer were titrated with defined concentrations of unlabeled proteins in the same buffer. Changes in fluorescence polarization were fit using a single binding model in the program Origin (OriginLab, Northampton MA).

### Electrostatic-driven docking

Fragments of β spectrin were modeled using Phyre2 [52] and Modeller [53]. Electrostatic potentials were determined solving the non-linear Poisson-Boltzman equation with PQR [54] and APBS [55] using a grid size of 2 Å, a salt concentration of 50 mM and a solvent radius of 1.4 Å, and protein accessible surface meshes were created using Chimera [56] with a default probe radius of 1.4 Å and vertex density of 2 per Å^2^. The electrostatic potential at each mesh grid point was interpolated using the gridData python module from MDAnalysis [57]. Grid points were filtered for potential values above +10 kT/e or below -10 kT/e and clustered using the DBSCAN algorithm [58] with an epsilon cut-off of 3 Å. The center of each cluster was determined and clusters were plotted using Matplotlib [59] as a function of distance from the protein N-terminus. The size of each cluster is proportional to the size of charged surface area. The central position of residues responsible for charged clusters in the protein was extrapolated on a grid mesh representing the protein surface. Clusters were drawn between highly solvent exposed residues less than ~10 Å apart.

The electrostatic charge distribution of KAHRP 5´ repeat was used to filter through possible paths on the spectrin surface. As the KAHRP 5´ repeat sequence elements have alternating charge (S3D and S3E Fig) paths on the spectrin surface were required to transverse between positive and negative clusters. Truncation of either KAHRP K2-4 or β10–14 resulted in reduced affinity, hence it was assumed that the entirety of these regions is needed for maximal binding, requiring KAHRP to have an extended binding configuration. As the distance between positively and negatively charged regions of KAHRP is greater than ~10 Å, but less than ~30 Å, assuming an extended protein conformation, we selected for possible spectrin surface paths featuring distances greater than 10 Å, but less than 30 Å, between positive and negative patches in Euclidean space. As the length of the KAHRP 5´ repeat is proportional to that of spectrin β10–14, and removal of any spectrin domains reduces KAHRP affinity, we restricted the possible paths on the spectrin surface to those that do not back-track but instead utilize as many spectrin domains as possible.

All possible spectrin surface paths meeting these conditions were found using the NetworkX [60] implementation of the Dijkstra’s shortest path algorithm and added to a directional network graph. In order to determine all paths from the spectrin N-terminus to the C-terminus an initial and a terminal node was added to the graph. The initial node was connected to all charged cluster centers less than 30 Å from the spectrin N-terminus, whereas the terminal node was connected to all charged cluster centers less than 30 Å from the spectrin C-terminus. All spectrin surface paths between the initial and the terminal node comprising more than 400 intermediate nodes for β10–14 or 100 intermediate nodes for β12–14 were found and the electrostatic potential along these paths determined using interpolation in Griddata package [61]. This typically resulted in ~2000 slightly different trajectories that were scored against the electrostatic profile of the KAHRP 5´ repeat, its truncations and scrambled sequences. As we have no information on KAHRP side chain orientation, the charge along the KAHRP backbone was set to +1 for Arg and Lys, -1 for Glu and Asp and 0 for all other residues, and the distance between residues set as the distance between adjacent C_α_ atoms in an extended protein conformation (3.8 Å). The backbone charge of KAHRP was then compared to the electrostatic charge of the surface path along spectrin using overlapped windows offset by 15.2 Å, and scored as follows:

- if the charges are opposite, -1 is added to the score.
- if the charges are opposite, and the absolute electrostatic potential of spectrin is greater than 10, a further -1 is added to the score.
- if the charges are like, +1 is added to the score.
- if the charges are like, and the absolute electrostatic potential of spectrin is greater than 10, a further +1 is added to the score.

For the β-catenin–Tcf complex ([62]; PDB ID 1G3J) part of the crystallographic structure shows evidence of electrostatic-driven binding; specifically, residues 10–29 of the Tcf peptide, which include nine acidic and two basic amino acids, and 251–583 of β-catenin. This region of the complex was used for benchmarking the ability of the electrostatic docking tool to *ab initio* predict a complex conformation (S5 Fig). The docking protocol was similar to that described for KAHRP–β spectrin above. As the Tcf peptide is short and lacks well defined charge repeats docking paths were not required to pass through alternating charge clusters. This resulted in 4400 unique paths that extensively covered the surface of β-catenin. These paths were scored assuming an anti-parallel β-catenin–Tcf orientation resulting in predominantly favorable docking scores.

### Molecular dynamics simulations

An initial model of the KAHRP–spectrin β10–14 complex was calculated using XPLOR-NIH [63]. NOE-like distance restraints were applied between KAHRP and spectrin β10–14 residues, defining the surface path derived by electrostatic-driven docking. Similar restraints were enforced within spectrin β10–14 to limit the conformational space of the spectrin backbone. The complex was further restrained by a potential of mean force that conducts a free-search for putative hydrogen bonds during the simulation and optimizes the spatial arrangement of peptidyl backbone units [64], and a conformational database potential [65]. The lowest energy structure from an ensemble of docked conformations thus generated was used to set up a 50 ns molecular dynamics (MD) simulation using the all atom force field AMBER99SB-ILDN [66] with TIP3P water. An ionic concentration of 50 mM NaCl and temperature of 298 K were used to replicate experimental conditions. Positional restraints were placed on the C_α_ atoms of spectrin to prevent it traversing the boundaries of a rectangular simulation box. Simulations were performed in a box 3 nm bigger than each spectrin dimension, pressure was maintained using the Parrinello-Rahman barostat [67] and temperature was maintained using the V-rescale thermostat [68]. All trajectories were generated and analyzed with GROMACS v5.1 [69]. The number of salt bridges was determined with VMD based on an oxygen to nitrogen distance cutoff of 4.5 Å [70, 71].

Triplicate MD simulations of the ATS-Core PFF_0845c –spectrin a17 complex were initiated from the two binding configurations predicted by HADDOCK. Simulations lasted 100 ns and were conducted in explicit water at 298 K with 50 mM NaCl. The all atom force field AMBER99SB-ILDN [66] with TIP3P water was used. Pressure was maintained using the Parrinello-Rahman barostat [67] and temperature was maintained at 298 K using the V-rescale thermostat [68]. Simulations were run and analyzed using GROMACS v4.6 [69]. Control simulations were also performed for ATS-Cores PFF_0845c and PF08_0141, as well as spectrin domain α17.

### NMR data collection and analysis, and NMR-driven docking

Sequence-specific resonance assignments of ATS variant PF08_0141 have been reported previously [31]. NMR experiments were performed using Bruker Avance II- or Avance III spectrometers with cryogenic TCI probeheads and magnetic field strengths 11.7 T, 14.1 T or 17.6 T. Samples were at 25°C and analysis buffer supplemented with 5% v/v D_2_O, 0.02% w/v NaN_3_ and 50 μM 4,4-dimethyl-4-silapentane-1-sulfonic acid unless otherwise noted. Sequence-specific resonance assignments were performed using 3D CBCA(CO)NH, CBCANH, HNCO, HN(CA)CO and HBHA(CO)NH pulse sequences. NMR data were processed using NMRpipe [72] and analyzed using CCPN Analysis [73]. Spectra overlays were prepared with Sparky [74]. Resonance perturbations were mapped using ^15^N-HSQC and 3D HNCO experiments, and perturbations from multiple nuclei types were combined using a sum of absolute differences approach weighted by nuclei-specific factors [75]. The spectrin α17 binding of substitution variants of ATS-Core PFF0845c was assessed using ^15^N-HSQC titrations with 50 μM ^15^N-labeled α17 and 0, 75, 150, 300 and 500 μM of unlabeled ATS-Core. Resonance perturbations of the eight most affected α17 peaks were globally fit to extract a single K_d_. Shown in Fig 6B are the average normalized perturbations of these eight peaks.

For NMR-driven docking the structure of ATS-Core variant PFF_0845c was modeled [53] using the highly similar ATS-Core variant PF08_0141 structure as template [31]. Prior to docking with HADDOCK [34] the solvent exposed surface areas of spectrin domain α17, derived from the spectrin α16–17 crystallographic structure, and ATS-Core PFF_0845c were determined with POPS [76]. All residues with solvent exposed surface area greater than 50 Å^2^ and combined NMR resonance perturbations greater than the mean were defined as active. Residues surrounding active amino acids were defined as passive. Two possible binding configurations were predicted by HADDOCK.

### Crystallization, X-ray data collection and analysis

Diffracting crystals of spectrin α16–17 were obtained using the sitting drop vapor diffusion technique at 4°C. A Mosquito robot (TTP LabTech, Melbourn UK) was used to setup 200 nl-size drops with 1:1 volume ratio of protein to mother liquor. Spectrin α16–17 at 4.3 mg/ml concentration formed diffracting crystals when mixed with 0.03 M MgCl_2_, 0.03 M CaCl_2_, 0.1 M 2-(*N*-morpholino)ethanesulfonic acid / imidazole pH 6.5 buffer, 20% v/v ethylene glycol and 10% w/v polyethylene glycol 8000. Rod crystals developed in 5 days. Crystals were flash frozen in liquid nitrogen and data were collected to 1.54 Å at the Diamond Light Source (DLS, Harwell UK) beamline I04-1. The space group was determined as P 2_1_ 2_1_ 2_1_ with one spectrin α16–17 molecule per asymmetric unit. Data were processed with XIA2 [77], analyzed by CCP4 [78], and the structure was solved by molecular replacement using Bables [79] and Phaser [80]. Model building was performed in Coot [81]. Iterative refinement was performed with Phenix [82] and Buster-TNT [83] using automatic TLS restraints. Crystallographic data collection and refinement statistics are provided in Table 2. Model quality was accessed with MolProbity [84]. Models were visualized using PyMOL [85] and analyzed using Dali [86].

**Table 2:** Crystallographic data and refinement statistics for α16–17.

PDB ID code	5J4O
***Data collection statistics***	
Space group	P 2_1_ 2_1_ 2_1_
Unit cell (Å)	a = 37.64, b = 43.67, c = 154.28
Beamline	DLS/I04-1
Wavelength (Å)	0.91741
Resolution range(Å)	38.57–1.54
High resolution shell (Å)	1.58–1.54
R_merge_^a^	0.065 (0.926)
R_pim_^a^	0.033 (0.442)
Completeness^a^ (%)	99.6 (99.2)
Multiplicity^a^	6.4 (6.2)
Mean I/σ(I)^a^	13.1 (1.7)
CC-half	0.998 (0.656)
***Refinement statistics***	
R_work_ (reflections)	20.9% (36601)
R_free_ (reflections)	22.9% (1880)
***Number of atoms***	
Protein (excluding H)	1887
Water	250
Ligands	28
***Average B factors (Å***^***2***^***)***	
Protein (excluding H)	34.9
Water	41.0
Ligands	69.4
***RMSD from ideal values***	
Bonds (Å)	0.01
Angles (°)	0.9
***Molprobity statistics***^b^	
Ramachandran favored (%)	99.57
Ramachandran disallowed (%)	0.00
Favored rotamers (%)	99.51
Poor rotamers (%)	0.00
Clashscore (percentile)^c^	0.26 (100^th^)
MolProbity score (percentile)^c^	0.60 (100^th^)

^a^ By Aimless [87], values in parentheses correspond to the high resolution shell

^b^ From MolProbity [84]

^c^ 100^th^ percentile is the best among structures of comparable resolution; 0^th^ percentile is the worst

### Accession numbers

Sequence-specific NMR assignments have been deposited in BioMagResBank under accession numbers 26772 and 26773 for the ATS-Core variant PFF0845c and spectrin α17, respectively. The model of spectrin α16–17 and associated crystallographic data have been deposited in the RCSB Protein Data Bank under accession number 5J4O.

## Supporting information

S1 Fig KAHRP is predicted to be disordered. Prediction of disorder propensity from the KAHRP amino acid sequence (UniProt Q9TY99) using the RONN [88], VL3 [89] and VSL2B [90] disorder prediction servers. The 50% threshold of disorder probability is shown as red line. A schematic representation of KAHRP is provided below as reference.
(TIF)

S2 Fig KAHRP K2 does not interact with spectrin α4. Shown here is an overlay of ^15^N-HSQC spectra of ^15^N-labeled 100 μM spectrin α4 alone (red) and in the presence of equimolar amounts of unlabeled KAHRP K2 (blue). The lack of significant perturbations in the NMR spectra suggests these protein constructs do not interact strongly.
(TIF)

S3 Fig KAHRP–spectrin binding assays. (A) FP titrations of labeled KAHRP K2 sub-fragments with spectrin β10–14 (default) or β12–14 (single data series with K2-4). Shown here are representative data from two independent experiments. Error bars indicate one standard deviation and derive from four technical repeats. Solid lines represent fits to single site binding models. (B) Similar titrations of KAHRP K2 with spectrin β10–14 sub-fragments. (C) Table of K_d_ constants derived from fits in (A) and (B) with schematic representations of protein constructs used. Green boxes correspond to KAHRP 5´-repeat elements, as shown in panels D and E. Grey boxes correspond to spectrin triple helical bundles. NA denotes titration data series for which good fit was not possible. Note that the apparent KAHRP–β10–14 affinity increases (K_d_ values decrease) as KAHRP fragments become smaller due to the reduction in entropic penalty associated with binding of smaller peptides. (D) Alignment (top) and sequence consensus (bottom) of the KAHRP 5´ sequence repeats. (E) Formal charge distribution of the 5´-repeat region of KAHRP derived from the amino acid sequence. The repeat boundaries are indicated. (F) FP titrations of labeled KAHRP K2 with spectrin β10–14 under increasing NaCl concentrations, showing decreased interaction affinity at higher ionic strengths. Shown here are representative data from two independent experiments. Error bars indicate one standard deviation and derive from five technical repeats. Solid lines represent fits to single site binding models. The interaction affinities are indicated.
(TIF)

S4 Fig Benchmarking of affinities predicted by the electrostatic docking tool. (A) Electrostatic docking score histograms of KAHRP fragments (K2-4, K2-5 and K2-6) with spectrin β10–14, and K2-4 with β12–14. (B) Table of lowest electrostatic docking scores and K_d_ values from FP titrations of labeled KAHRP and spectrin fragments. A correlation between docking scores and binding energies, ΔG, is shown in (C).
(TIF)

S5 Fig Benchmarking of complex conformations predicted by the electrostatic docking tool. For maximum similarity to the KAHRP–spectrin complex, we searched the protein data bank for a complex composed of a disordered protein interacting with a folded scaffold, and which has a binding interface with high charge density. (A) Section of the crystallographic structure of the β-catenin–Tcf complex ([62]; PDB ID 1G3J) with the highest proportion of electrostatic interactions, which was used in benchmarking the electrostatic docking tool. β-catenin is shown in grey and Tcf in cyan. The protein N- and C-termini are indicated. (B) Solvent accessible surface area of β-catenin, with (top) positively charged residues in blue and negatively charged residues in red in two opposing orientations. (Bottom) Similar representation of β-catenin with positive (blue) and negative (red) charge clusters used in calculating possible docking paths. (C) Representative docking paths generated by the electrostatic docking algorithm. As seen, paths extensively cover the β-catenin surface. (D) The lowest energy (docking score) predicted conformation (path) of Tcf (orange) is shown overlaid to the crystallographic structure of the β-catenin–Tcf complex (grey and cyan). The docked conformation of Tcf follows the same trajectory as the Tcf peptide in the high-resolition structure. (E) Histogram of the docking (charge complementation) score calculated for all putative β-catenin–Tcf paths. (F) Graph of the electrostatic potential of the β-catenin surface along the length of the lowest scoring path (kT/e, grey), overlaid with the formal charge of the Tcf peptide (orange). The relative protein positions along the docking path are denoted using a distance calculated from the N-terminus of β-catenin.
(TIF)

S6 Fig MD simulations of the KAHRP 5´ repeat–β10–14 complex. (A) Root mean square deviation (RMSD) in the position of C_α_ atoms of the KAHRP 5´-repeat bound to spectrin β10–14 during triplicate 50 ns MD simulations. Plotted here is the RMSD from starting coordinates derived by placing the KAHRP 5´-repeat along the lowest docking score path on spectrin β10–14. RMSD plateauing indicates convergence of the simulation. (B) Number of electrostatic interactions (salt-bridges) formed between KAHRP and spectrin residues during the MD simulations. (C) Detail from the MD simulation of the KAHRP–spectrin complex. The spectrin is shown in surface representation colored by electrostatic potential; KAHRP residues are shown as sticks. A number of favorable electrostatic interactions can be seen. (D) Overlays of KAHRP 5´ repeat (red) conformations and spectrin β10–14 (blue) in MD simulations. KAHRP conformations were extracted every 1 ns during 50 ns of simulation time. Top and bottom correspond to two replicate simulations showing that KAHRP remains bound to β10–14, albeit with substantial dynamicity that results in alternative conformations being adopted by the KAHRP C-terminus during the second simulation.
(TIF)

S7 Fig Sequence specificity of the KAHRP–spectrin interaction. (A) Formal charge distribution of sequence scrambled peptides compared to the canonical KAHRP 5´ repeat. (B) Histograms of electrostatic docking scores for sequence scrambled peptides with spectrin β10–14. Filled bars correspond to docking scores with proteins in antiparallel orientations; black lines denote scores of parallel orientations. (C) FP titrations of labeled KAHRP 5´ repeat (K2-4) or sequence scrambled peptides with unlabeled spectrin β10–14. Shown here are representative data from two independent experiments. Error bars indicate one standard deviation and derive from four technical repeats. The electrostatic docking scores and K_d_ values are shown.
(TIF)

S8 Fig ATS–spectrin binding assays. (A) FP titrations of labeled ATS from *Pf*EMP1 variant PF08_0141 with unlabeled α and β spectrin fragments. Shown here are representative data from two independent experiments. Error bars indicate one standard deviation and derive from four technical repeats. Solid lines represent fits to single site binding models. K_d_ values are indicated. (B) Similar titrations of labeled ATS-NCore, comprising the ATS-N and ATS-Core regions, from *Pf*EMP1 variant PF08_0141 with unlabeled spectrin α17-C sub-fragments. (C) FP titrations of labeled ATS domains from *Pf*EMP1 variants with spectrin domain α17. Error bars indicate one standard deviation from three experimental repeats, each with four technical repeats.
(TIF)

S9 Fig NMR of ATS–spectrin binding. (A) ^15^N-HSQC spectra of 100 μM ^15^N-labeled ATS-N alone (red) or in the presence of equimolar concentration of unlabeled spectrin α17-C (blue). Spectra were recorded at 10°C. Reduction of NMR peak intensity in the presence of spectrin α17-C is indicative of binding. Similar spectra of ATS-Core (25°C, B), ATS-M (10°C, E) and ATS-C (10°C, F). Boxed areas are regions magnified in Fig 4B. (C, D) Relative NMR peak intensities from of ATS-N and ATS-Core, respectively, in the presence of spectrin α17-C. Intensities from overlapped NMR peaks were not included. The position at which *Pf*EMP1 segments are joined to form ATS-Core is indicated by a dashed line in the right graph. Error bars correspond to one standard deviation and derive from the spectral signal / noise ratio.
(TIF)

S10 Fig Crystallographic structure of spectrin α16–17. (A) Schematic representation in two orthogonal views of the 1.54 Å resolution erythrocytic spectrin α16–17 crystallographic structure. Spectrin repeat α16 (R16) is oriented towards the bottom, while α17 (R17) is at the top. Crystallographic data and refinement statistics are provided in Table 2. α17 displays a canonical spectrin repeat structure that superimposes well with previously resolved spectrin modules (≥1.5 Å C_α_ RMSD over the entire repeat). In contrast, α16 features uncommonly large bends in helices α2 and α3, indicated by red arrows, which result in substantially worse superposition of this domain with other spectrin repeats (≥2.5 Å C_α_ RMSD). (B) Superposition of the spectrin α16–17 structure (blue) with repeats 15–16 (wheat, PDB 1U4Q, [91]) and 16–17 (red, PDB 1CUN, [92]) of the chicken brain α spectrin. Superposition was performed along the first spectrin repeat in each case. 1U4Q displays a near-linear arrangement of spectrin repeats, 1CUN shows a ~18° angle between the two domains, while α16–17 shows a ~40° angle between domains as a result of bends in helices α2 and α3 (panel A). (C) Magnification of the area where helices α2 and α3 diverge between α16–17 (blue) and 1U4Q (wheat).
(TIF)

S11 Fig Docking and simulations of the ATS-Core–spectrin α17 complex. (A,B) Schematic representations, in two orthogonal views, of the two main complex conformations produced by docking ATS-Core PFF0845c to spectrin α17 using NMR peak perturbations as restraints. (C) RMSD in C_α_ atom positions of ATS-Core bound to spectrin α17 during triplicate MD simulations. Plotted here is the RMSD from the complex conformation 1 or 2 as function of simulation time. RMSD plateauing indicates convergence of the simulation. (D) Change in surface area buried at the ATS-Core / spectrin α17 interface during triplicate MD simulations starting with the complex conformation 1 or 2 coordinates. In all metrics of panels C and D, MD simulations starting from conformation 1 show smaller divergence over time (less RMSD, maintenance of buried surface area) compared to those from conformation 2.
(TIF)

S1 Software Electrostatic docking software tool. Provided here is a compressed file that includes all scripts used for the calculation of docking conformations between two charged proteins. The specific examples of docking the KAHRP 5´ repeat to spectrin β10–14, and predicting the β-catenin–Tcf complex, are shown in separate directories. A README file with simple instructions and software dependencies is also included.
(ZIP)
