# Supplementary figures and images for: Structural analysis of P. falciparum KAHRP and PfEMP1 complexes with host erythrocyte spectrin suggests a model for cytoadherent knob protrusions
Source: PLoS Pathog. 2017 Aug 14;13(8):e1006552. doi: 10.1371/journal.ppat.1006552 (PMC5570508; doi:10.1371/journal.ppat.1006552)

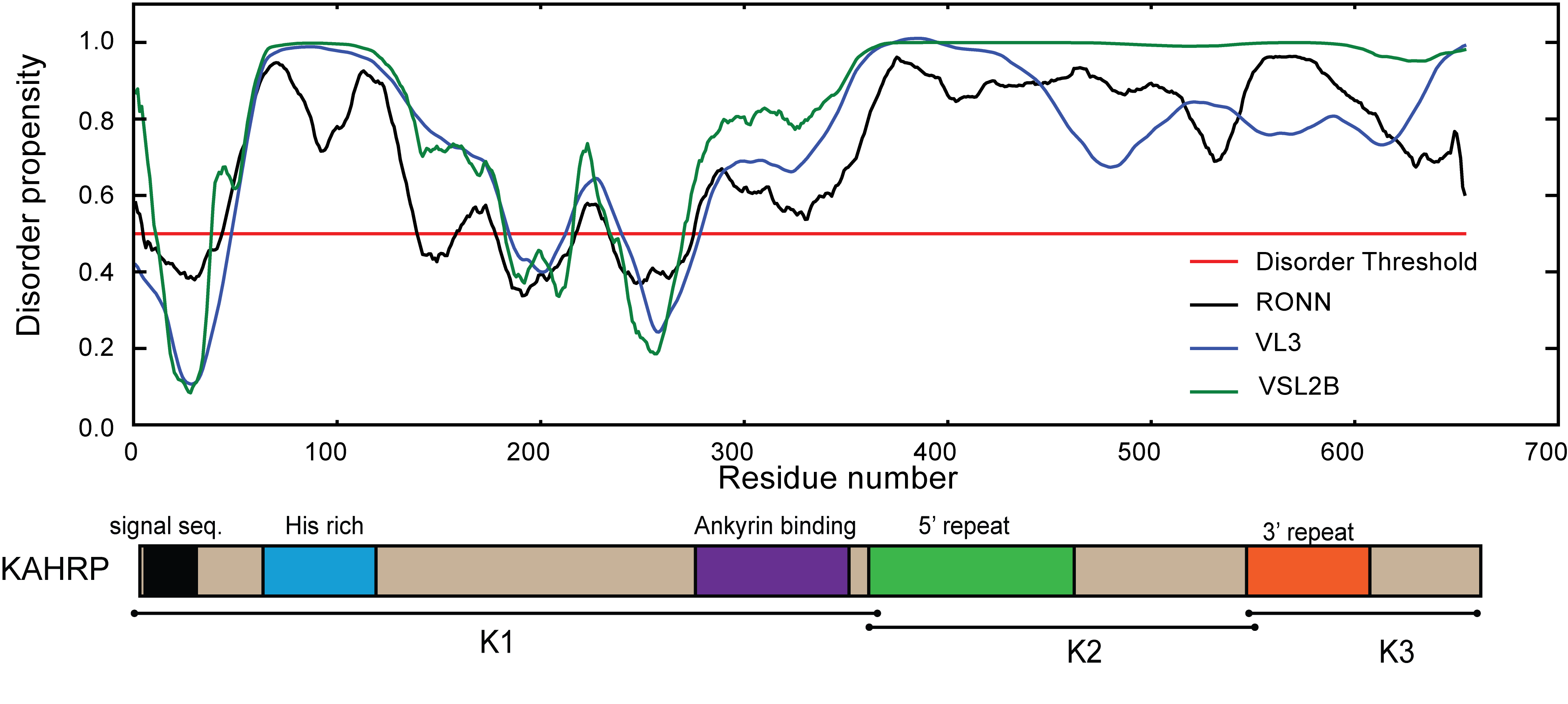

Supplement: S1 Fig — Prediction of disorder propensity from the KAHRP amino acid sequence (UniProt Q9TY99) using the RONN [88], VL3 [89] and VSL2B [90] disorder prediction servers. The 50% threshold of disorder probability is shown as red line. A schematic representation of KAHRP is provided below as reference. (TIF) [file ppat.1006552.s001.tif]

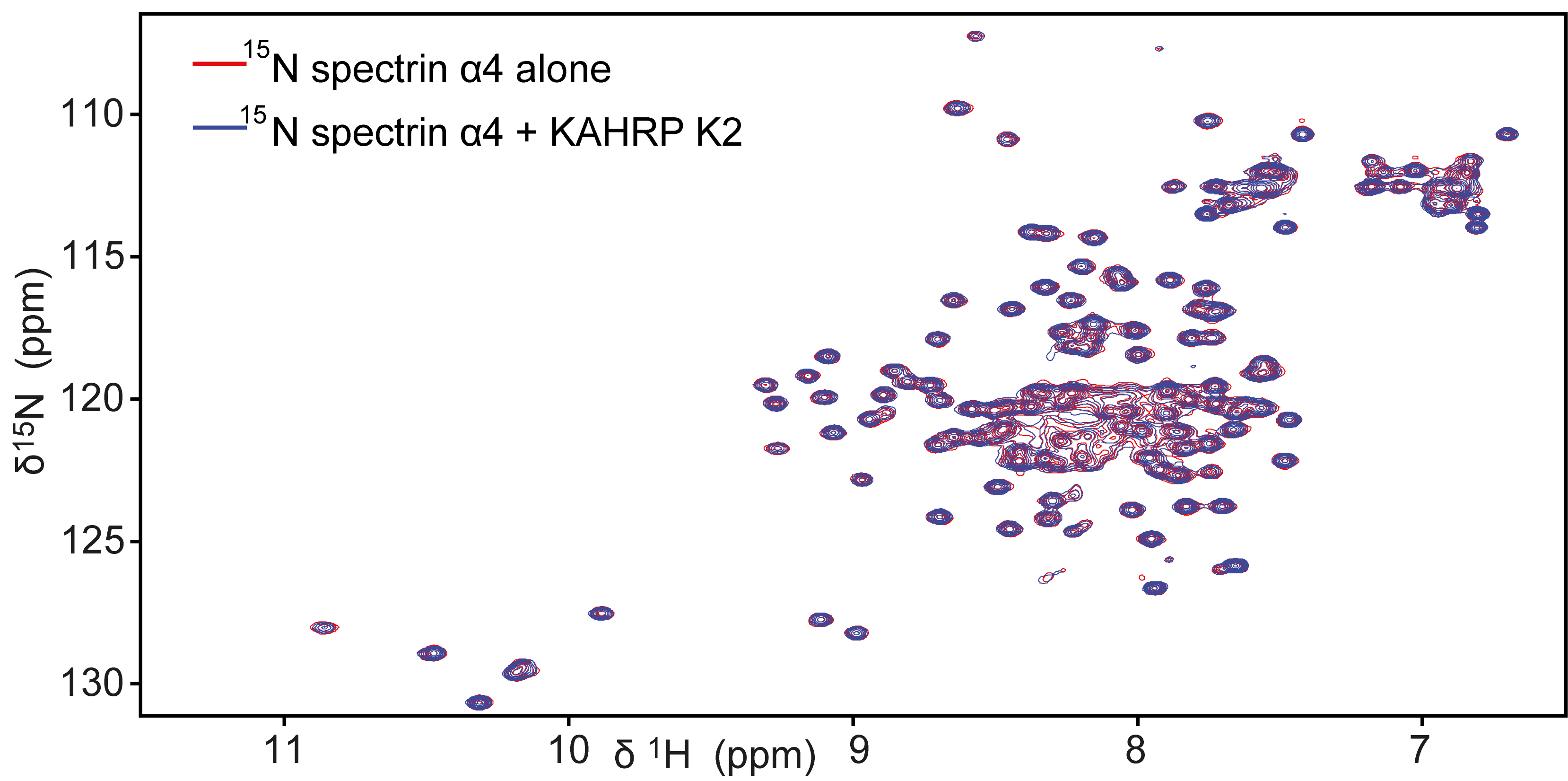

Supplement: S2 Fig — Shown here is an overlay of 15N-HSQC spectra of 15N-labeled 100 μM spectrin α4 alone (red) and in the presence of equimolar amounts of unlabeled KAHRP K2 (blue). The lack of significant perturbations in the NMR spectra suggests these protein constructs do not interact strongly. (TIF) [file ppat.1006552.s002.tif]

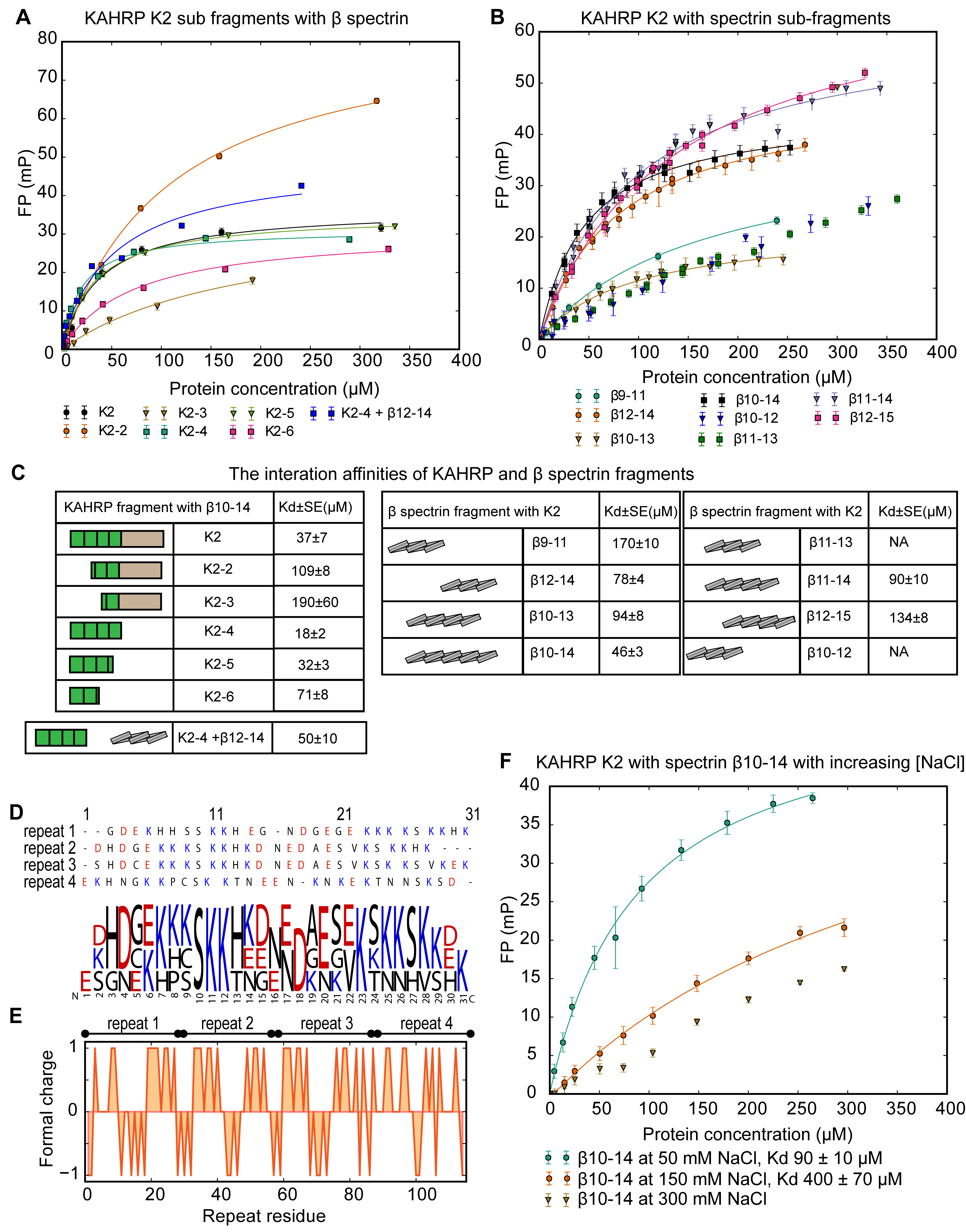

Supplement: S3 Fig — (A) FP titrations of labeled KAHRP K2 sub-fragments with spectrin β10–14 (default) or β12–14 (single data series with K2-4). Shown here are representative data from two independent experiments. Error bars indicate one standard deviation and derive from four technical repeats. Solid lines represent fits to single site binding models. (B) Similar titrations of KAHRP K2 with spectrin β10–14 sub-fragments. (C) Table of Kd constants derived from fits in (A) and (B) with schematic representations of protein constructs used. Green boxes correspond to KAHRP 5´-repeat elements, as shown in panels D and E. Grey boxes correspond to spectrin triple helical bundles. NA denotes titration data series for which good fit was not possible. Note that the apparent KAHRP–β10–14 affinity increases (Kd values decrease) as KAHRP fragments become smaller due to the reduction in entropic penalty associated with binding of smaller peptides. (D) Alignment (top) and sequence consensus (bottom) of the KAHRP 5´ sequence repeats. (E) Formal charge distribution of the 5´-repeat region of KAHRP derived from the amino acid sequence. The repeat boundaries are indicated. (F) FP titrations of labeled KAHRP K2 with spectrin β10–14 under increasing NaCl concentrations, showing decreased interaction affinity at higher ionic strengths. Shown here are representative data from two independent experiments. Error bars indicate one standard deviation and derive from five technical repeats. Solid lines represent fits to single site binding models. The interaction affinities are indicated. (TIF) [file ppat.1006552.s003.tif]

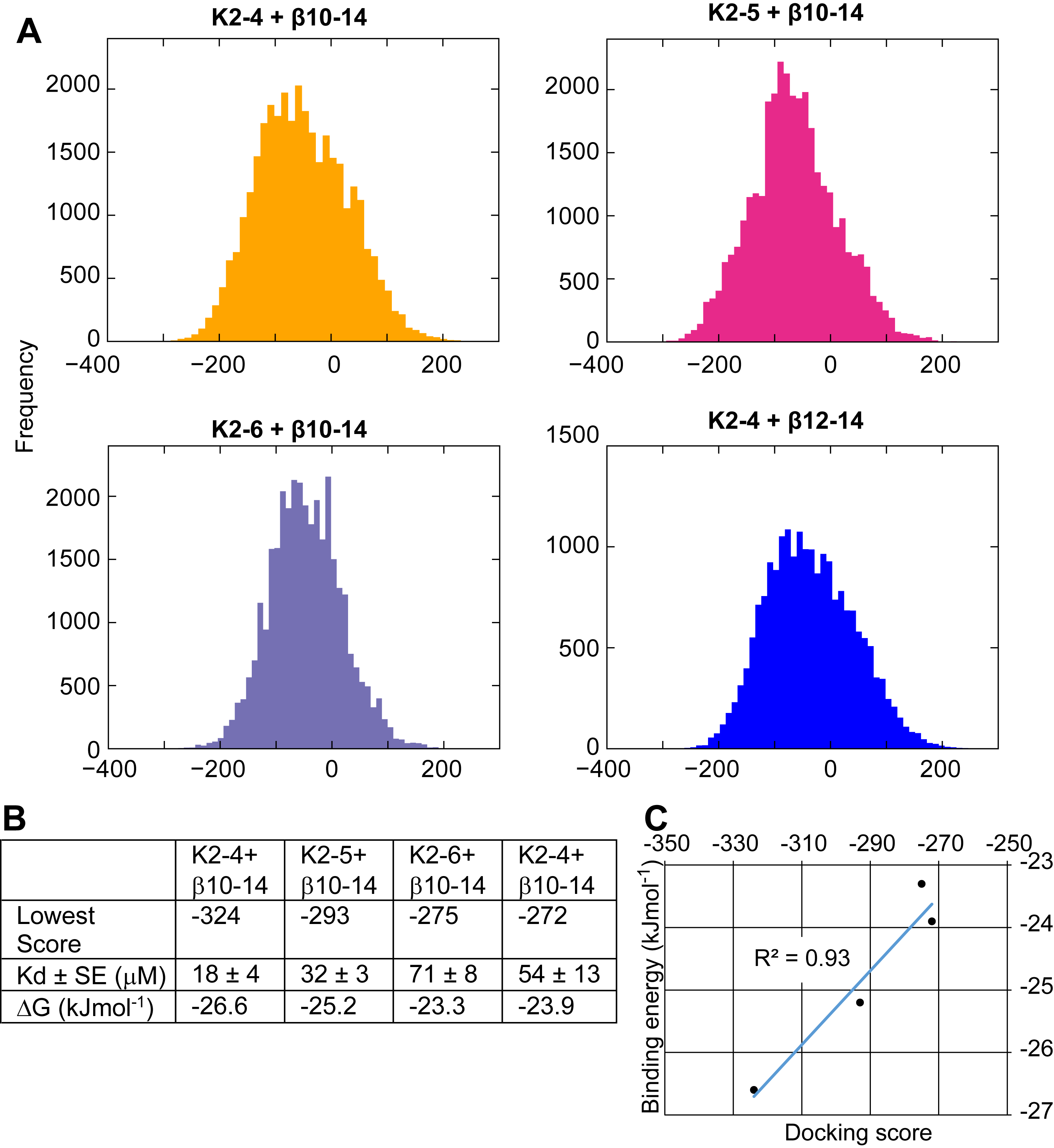

Supplement: S4 Fig — (A) Electrostatic docking score histograms of KAHRP fragments (K2-4, K2-5 and K2-6) with spectrin β10–14, and K2-4 with β12–14. (B) Table of lowest electrostatic docking scores and Kd values from FP titrations of labeled KAHRP and spectrin fragments. A correlation between docking scores and binding energies, ΔG, is shown in (C). (TIF) [file ppat.1006552.s004.tif]

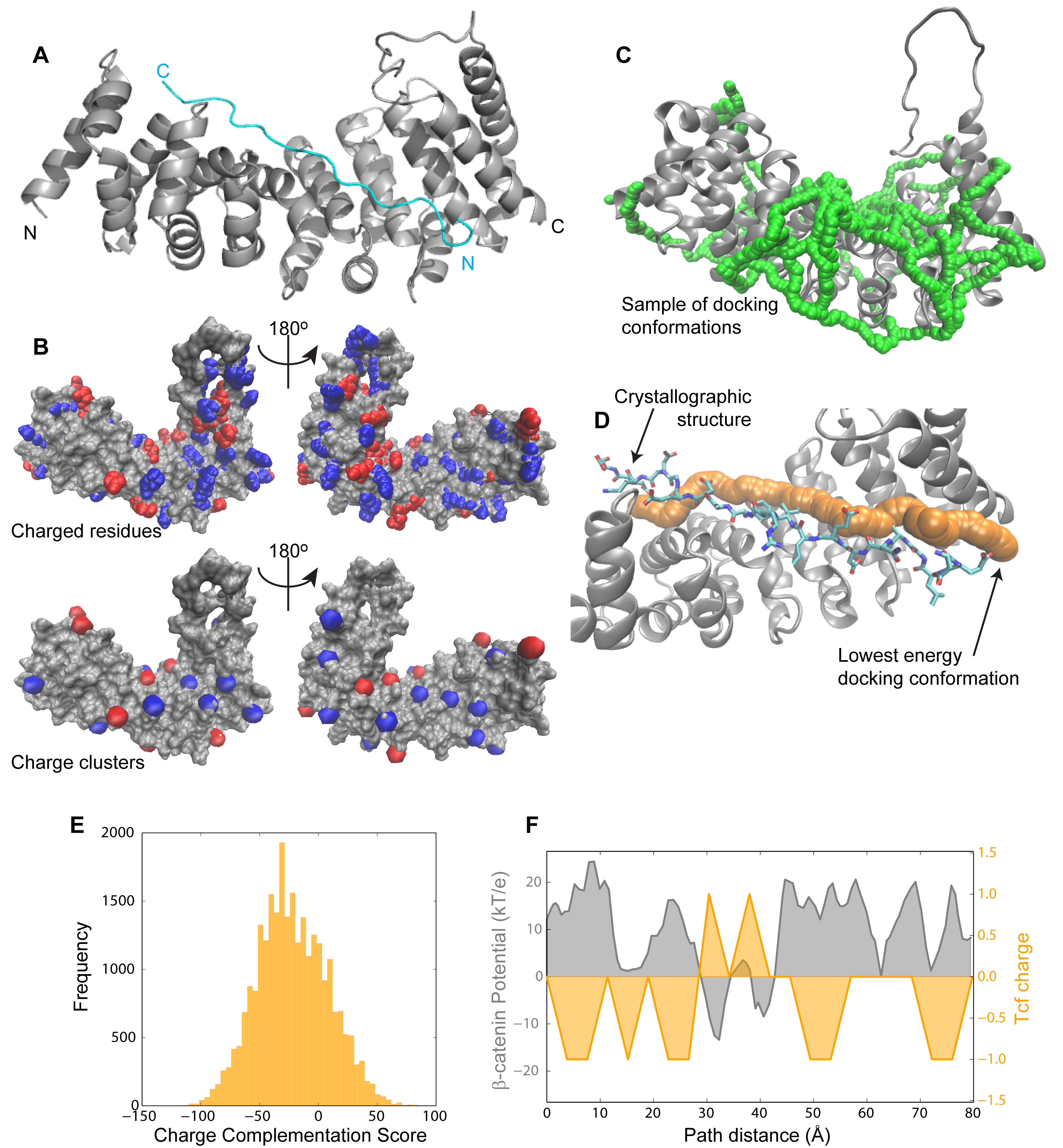

Supplement: S5 Fig — For maximum similarity to the KAHRP–spectrin complex, we searched the protein data bank for a complex composed of a disordered protein interacting with a folded scaffold, and which has a binding interface with high charge density. (A) Section of the crystallographic structure of the β-catenin–Tcf complex ([62]; PDB ID 1G3J) with the highest proportion of electrostatic interactions, which was used in benchmarking the electrostatic docking tool. β-catenin is shown in grey and Tcf in cyan. The protein N- and C-termini are indicated. (B) Solvent accessible surface area of β-catenin, with (top) positively charged residues in blue and negatively charged residues in red in two opposing orientations. (Bottom) Similar representation of β-catenin with positive (blue) and negative (red) charge clusters used in calculating possible docking paths. (C) Representative docking paths generated by the electrostatic docking algorithm. As seen, paths extensively cover the β-catenin surface. (D) The lowest energy (docking score) predicted conformation (path) of Tcf (orange) is shown overlaid to the crystallographic structure of the β-catenin–Tcf complex (grey and cyan). The docked conformation of Tcf follows the same trajectory as the Tcf peptide in the high-resolition structure. (E) Histogram of the docking (charge complementation) score calculated for all putative β-catenin–Tcf paths. (F) Graph of the electrostatic potential of the β-catenin surface along the length of the lowest scoring path (kT/e, grey), overlaid with the formal charge of the Tcf peptide (orange). The relative protein positions along the docking path are denoted using a distance calculated from the N-terminus of β-catenin. (TIF) [file ppat.1006552.s005.tif]

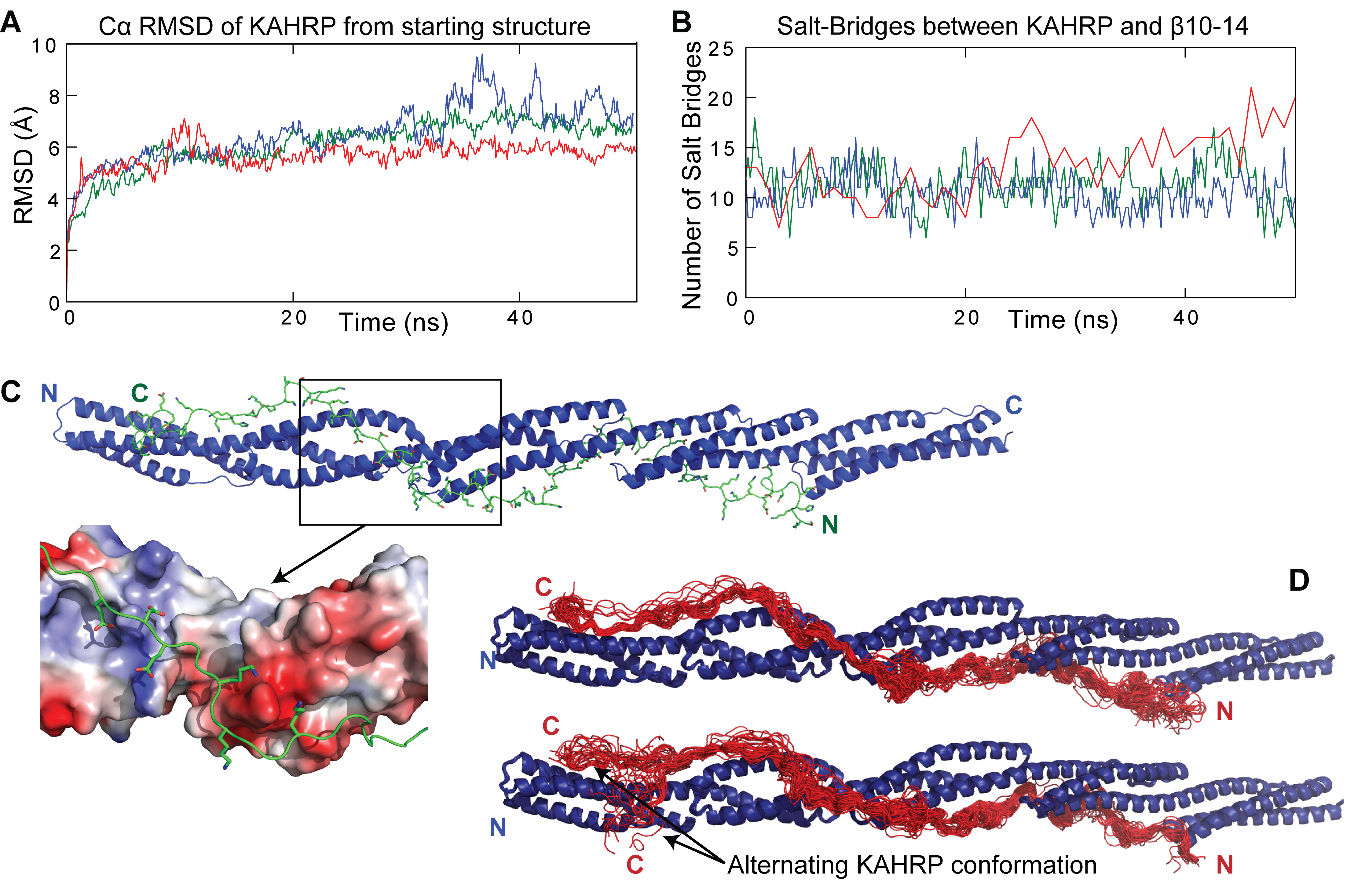

Supplement: S6 Fig — (A) Root mean square deviation (RMSD) in the position of Cα atoms of the KAHRP 5´-repeat bound to spectrin β10–14 during triplicate 50 ns MD simulations. Plotted here is the RMSD from starting coordinates derived by placing the KAHRP 5´-repeat along the lowest docking score path on spectrin β10–14. RMSD plateauing indicates convergence of the simulation. (B) Number of electrostatic interactions (salt-bridges) formed between KAHRP and spectrin residues during the MD simulations. (C) Detail from the MD simulation of the KAHRP–spectrin complex. The spectrin is shown in surface representation colored by electrostatic potential; KAHRP residues are shown as sticks. A number of favorable electrostatic interactions can be seen. (D) Overlays of KAHRP 5´ repeat (red) conformations and spectrin β10–14 (blue) in MD simulations. KAHRP conformations were extracted every 1 ns during 50 ns of simulation time. Top and bottom correspond to two replicate simulations showing that KAHRP remains bound to β10–14, albeit with substantial dynamicity that results in alternative conformations being adopted by the KAHRP C-terminus during the second simulation. (TIF) [file ppat.1006552.s006.tif]

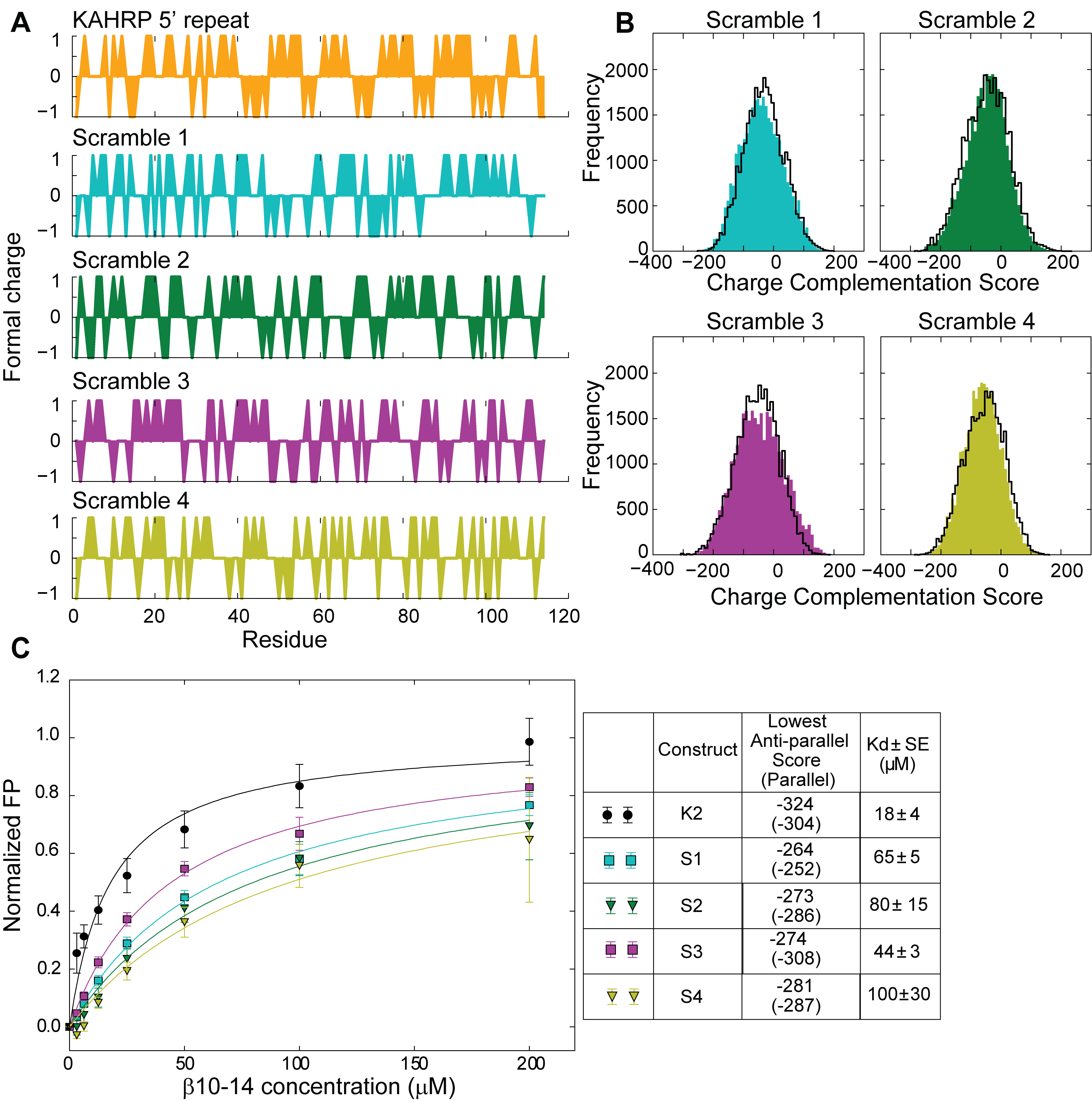

Supplement: S7 Fig — (A) Formal charge distribution of sequence scrambled peptides compared to the canonical KAHRP 5´ repeat. (B) Histograms of electrostatic docking scores for sequence scrambled peptides with spectrin β10–14. Filled bars correspond to docking scores with proteins in antiparallel orientations; black lines denote scores of parallel orientations. (C) FP titrations of labeled KAHRP 5´ repeat (K2-4) or sequence scrambled peptides with unlabeled spectrin β10–14. Shown here are representative data from two independent experiments. Error bars indicate one standard deviation and derive from four technical repeats. The electrostatic docking scores and Kd values are shown. (TIF) [file ppat.1006552.s007.tif]

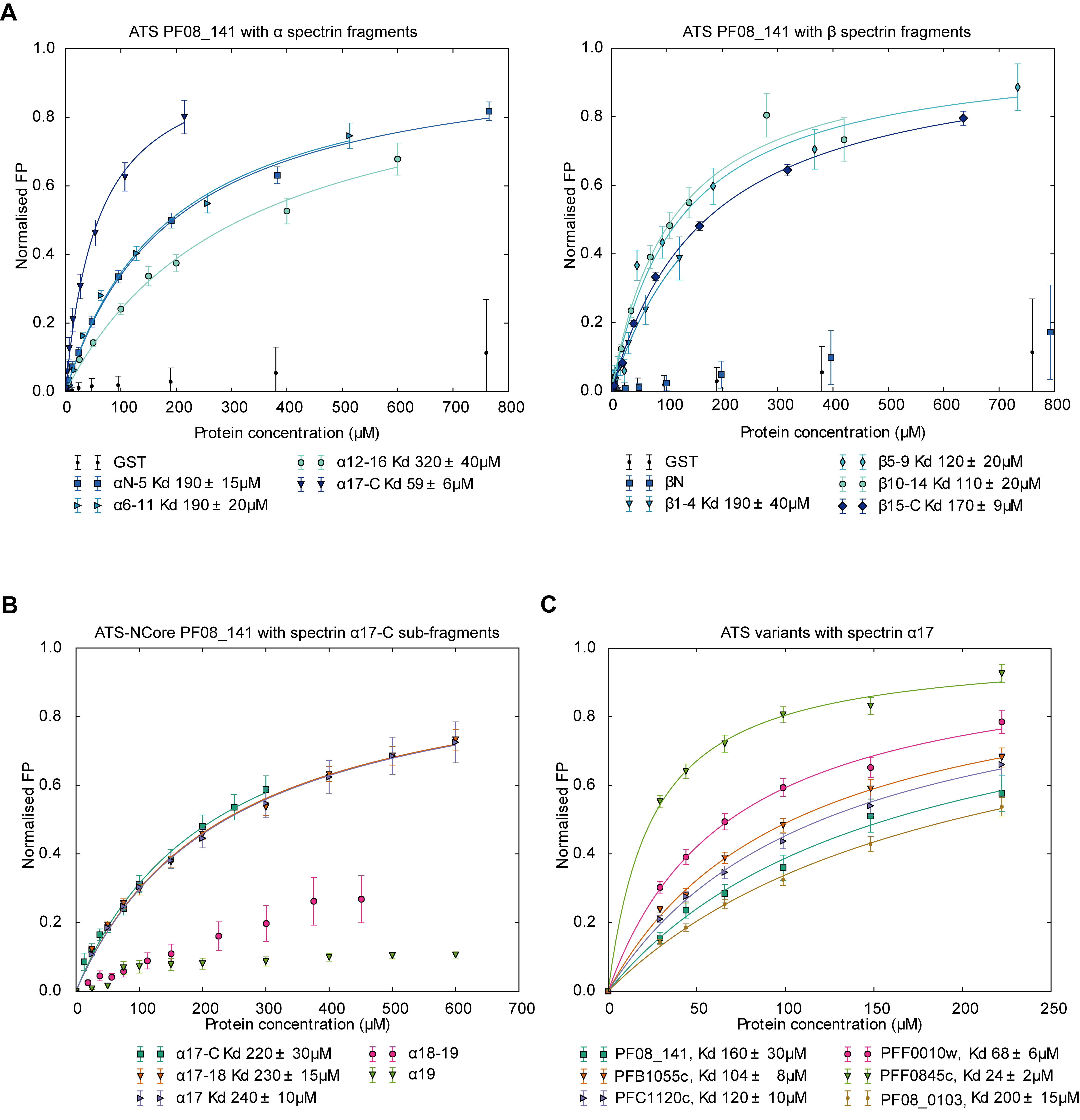

Supplement: S8 Fig — (A) FP titrations of labeled ATS from PfEMP1 variant PF08_0141 with unlabeled α and β spectrin fragments. Shown here are representative data from two independent experiments. Error bars indicate one standard deviation and derive from four technical repeats. Solid lines represent fits to single site binding models. Kd values are indicated. (B) Similar titrations of labeled ATS-NCore, comprising the ATS-N and ATS-Core regions, from PfEMP1 variant PF08_0141 with unlabeled spectrin α17-C sub-fragments. (C) FP titrations of labeled ATS domains from PfEMP1 variants with spectrin domain α17. Error bars indicate one standard deviation from three experimental repeats, each with four technical repeats. (TIF) [file ppat.1006552.s008.tif]

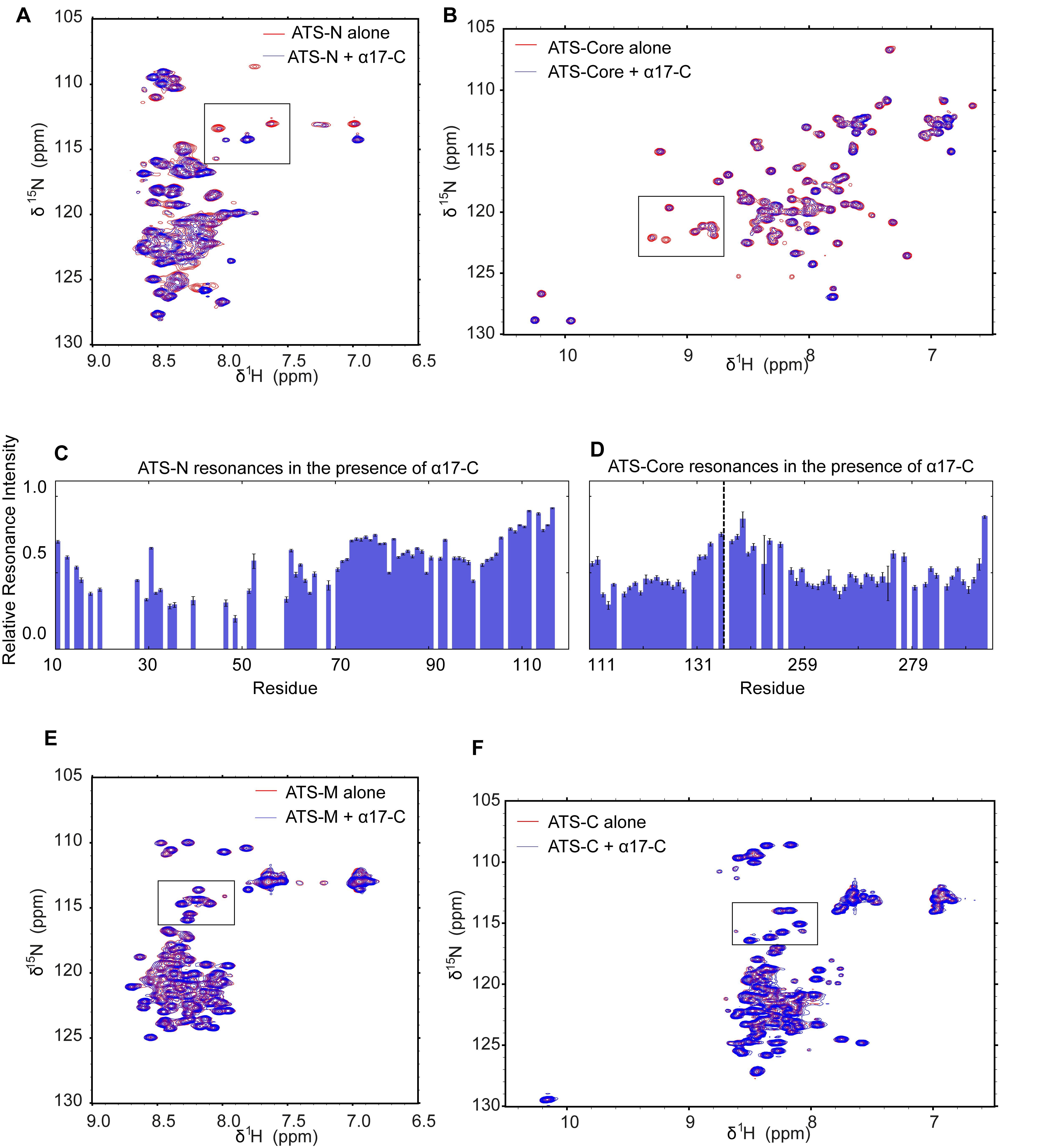

Supplement: S9 Fig — (A) 15N-HSQC spectra of 100 μM 15N-labeled ATS-N alone (red) or in the presence of equimolar concentration of unlabeled spectrin α17-C (blue). Spectra were recorded at 10°C. Reduction of NMR peak intensity in the presence of spectrin α17-C is indicative of binding. Similar spectra of ATS-Core (25°C, B), ATS-M (10°C, E) and ATS-C (10°C, F). Boxed areas are regions magnified in Fig 4B. (C, D) Relative NMR peak intensities from of ATS-N and ATS-Core, respectively, in the presence of spectrin α17-C. Intensities from overlapped NMR peaks were not included. The position at which PfEMP1 segments are joined to form ATS-Core is indicated by a dashed line in the right graph. Error bars correspond to one standard deviation and derive from the spectral signal / noise ratio. (TIF) [file ppat.1006552.s009.tif]

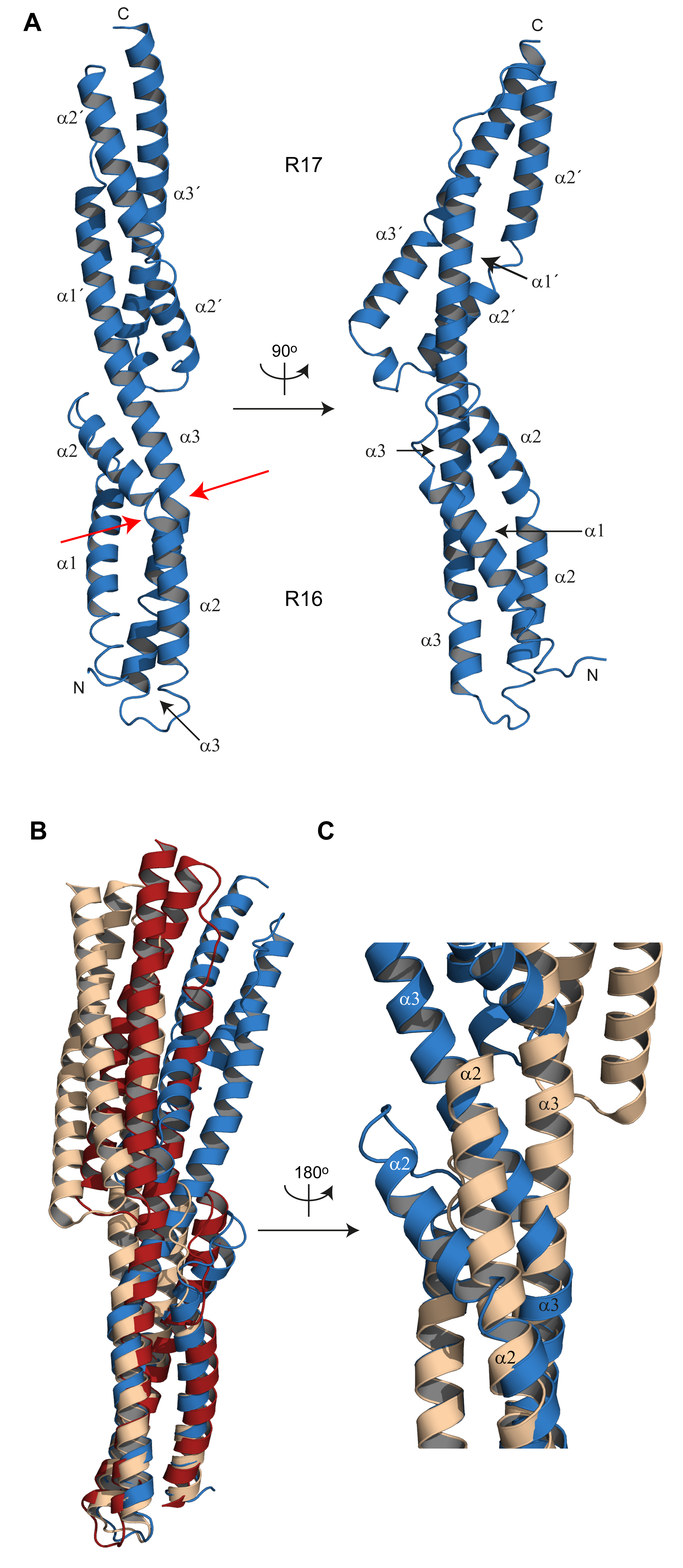

Supplement: S10 Fig — (A) Schematic representation in two orthogonal views of the 1.54 Å resolution erythrocytic spectrin α16–17 crystallographic structure. Spectrin repeat α16 (R16) is oriented towards the bottom, while α17 (R17) is at the top. Crystallographic data and refinement statistics are provided in Table 2. α17 displays a canonical spectrin repeat structure that superimposes well with previously resolved spectrin modules (≥1.5 Å Cα RMSD over the entire repeat). In contrast, α16 features uncommonly large bends in helices α2 and α3, indicated by red arrows, which result in substantially worse superposition of this domain with other spectrin repeats (≥2.5 Å Cα RMSD). (B) Superposition of the spectrin α16–17 structure (blue) with repeats 15–16 (wheat, PDB 1U4Q, [91]) and 16–17 (red, PDB 1CUN, [92]) of the chicken brain α spectrin. Superposition was performed along the first spectrin repeat in each case. 1U4Q displays a near-linear arrangement of spectrin repeats, 1CUN shows a ~18° angle between the two domains, while α16–17 shows a ~40° angle between domains as a result of bends in helices α2 and α3 (panel A). (C) Magnification of the area where helices α2 and α3 diverge between α16–17 (blue) and 1U4Q (wheat). (TIF) [file ppat.1006552.s010.tif]

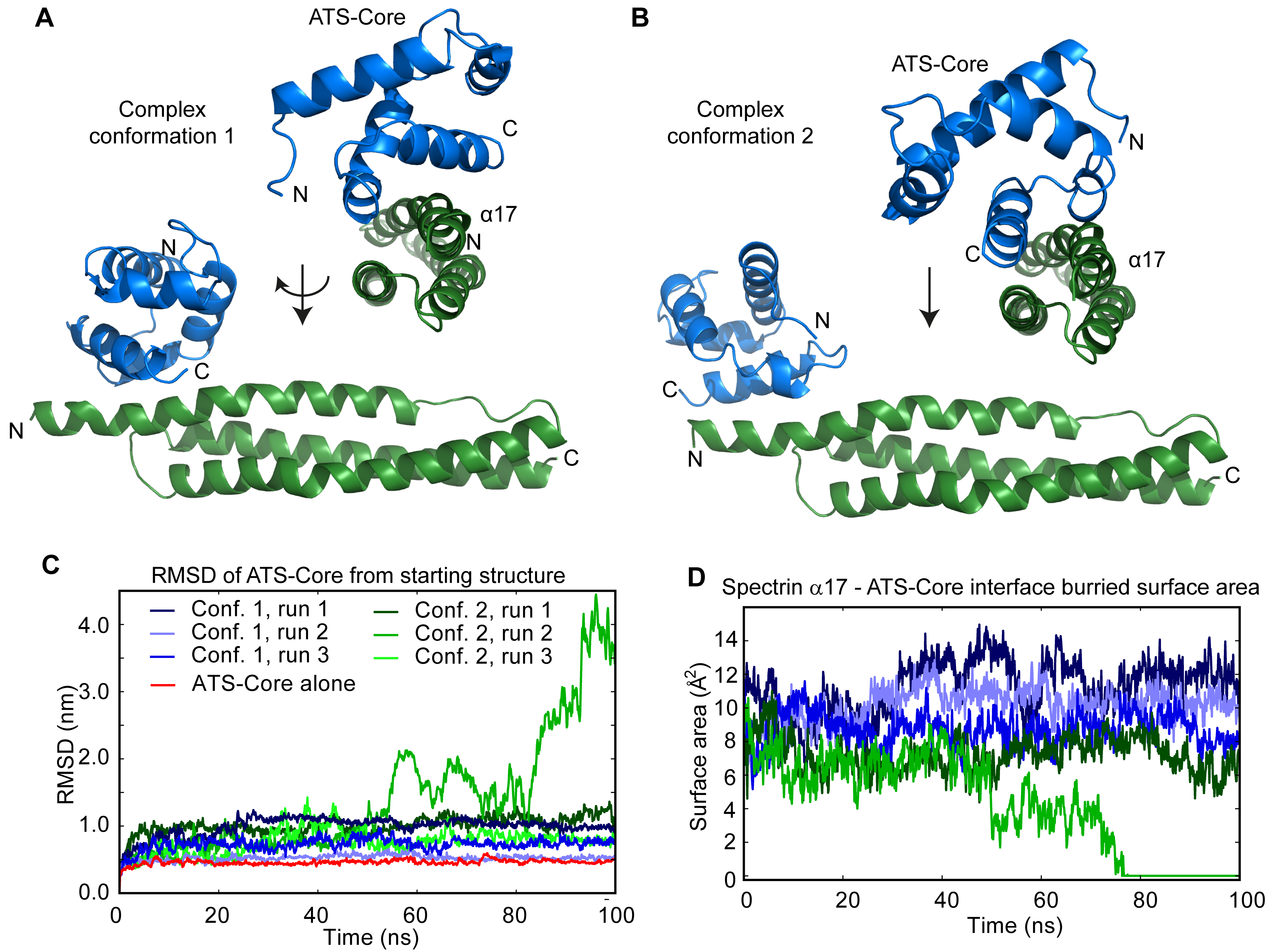

Supplement: S11 Fig — (A,B) Schematic representations, in two orthogonal views, of the two main complex conformations produced by docking ATS-Core PFF0845c to spectrin α17 using NMR peak perturbations as restraints. (C) RMSD in Cα atom positions of ATS-Core bound to spectrin α17 during triplicate MD simulations. Plotted here is the RMSD from the complex conformation 1 or 2 as function of simulation time. RMSD plateauing indicates convergence of the simulation. (D) Change in surface area buried at the ATS-Core / spectrin α17 interface during triplicate MD simulations starting with the complex conformation 1 or 2 coordinates. In all metrics of panels C and D, MD simulations starting from conformation 1 show smaller divergence over time (less RMSD, maintenance of buried surface area) compared to those from conformation 2. (TIF) [file ppat.1006552.s011.tif]
