# Supplementary material for: Structural analysis of P. falciparum KAHRP and PfEMP1 complexes with host erythrocyte spectrin suggests a model for cytoadherent knob protrusions
Source: PLoS Pathog. 2017 Aug 14;13(8):e1006552. doi: 10.1371/journal.ppat.1006552 (PMC5570508; doi:10.1371/journal.ppat.1006552)
Supplement: S1 Software — Provided here is a compressed file that includes all scripts used for the calculation of docking conformations between two charged proteins. The specific examples of docking the KAHRP 5´ repeat to spectrin β10–14, and predicting the β-catenin–Tcf complex, are shown in separate directories. A README file with simple instructions and software dependencies is also included. (ZIP) [file ppat.1006552.s012.zip › scripts/read_me.rtf]

Docking involves three parts: path finding, path scoring, co-ordinate output. For the two examples given, beta spectrum repeats 10-14 and benchmarking, each step has been separated into folders. Some out put files produced are very large. All out put should be able to be generated by following the protocols, and truncated output has been provided for out.dx, paths.txt and points.txt in the path finding folder such that results can be checked.Software requirements:The script is written in python; you will require numpy, gridData (https://github.com/MDAnalysis/GridDataFormats), networkx (https://networkx.github.io/). In addition, pqr, apbs, chimeria are needed for file preparation.Path findingSet up:Open pdb in chimera, display surface and export scene to out.obj, this file carries the surface description as a vector object. Use pqr to generate out.pqr. Use apbs, with out.pqr and apbs.in to generate out.dx, this carries the electrostatic data in grid data format. See http://www.poissonboltzmann.org/docs/calculating/ Finally, determine which residues define the charged patches and put the average coordinate into a text file (posneg.txt for bench marking or pos_filtered.txt and neg_filtered.txt for beta10-14).Run:python all_paths.py> paths.txtTakes as inputs: 	The surface: out.obj	Positive and negative coordinates: posneg.txt or pos_filtered.txt and neg_filtered.txt	The electrostatic potential file: out.dxOutputs: 	distance along path and potential by dumping text (paths.txt)    cartesian coordinates output to a file (points.txt)ScoringRun:python score.pyTakes as input:	Paths finding that has been transposed, commas and brackets removed and zero filled so each column is the same length: paths.txt	Charge versus distance for the peptide: peptide.txt	A vector “shift” is supplied in the script for allowing a set of off-sets between the path and the peptideOutputs:	All scores into a file: all_score.txt	Best score, the path and shift index.Use the output to extract the best path and save it as:	best_path.txtCoordinate findingrun:python coords_search.py>coord_out.txtInput:	Takes the coordinates of the best path: best_path.txt	The pdb file: model.pdb	The list of paths: paths.txt	The charge along the peptide: peptide.txtoutputs:	The atom of the pdb file that each residue of the peptide is closest to when aligned to the best electrostatic path.
